# Ocular mucosal homeostasis of teleost fish provides insight into the coevolution between microbiome and mucosal immunity

**DOI:** 10.1186/s40168-023-01716-6

**Published:** 2024-01-13

**Authors:** Weiguang Kong, Gaofeng Cheng, Jiafeng Cao, Jiaqian Yu, Xinyou Wang, Zhen Xu

**Affiliations:** 1 Key Laboratory of Breeding Biotechnology and Sustainable Aquaculture, Institute of Hydrobiology, Chinese Academy of Sciences, Wuhan, 430072 China; 2 Department of Aquatic Animal Medicine, College of Fisheries, Huazhong Agricultural University, Wuhan, 430070 Hubei China; 3 Laboratory of Biochemistry and Molecular Biology, School of Marine Sciences, Meishan Campus, Ningbo University, Ningbo, 315832 China; 4 Liaoning Key Laboratory of Marine Animal Immunology, Dalian Ocean University, Dalian, 116023 China

**Keywords:** Ocular microbiota, Evolution, Mucosal immunity, IHNV infection, Rainbow trout

## Abstract

**Background:**

The visual organ plays a crucial role in sensing environmental information. However, its mucosal surfaces are constantly exposed to selective pressures from aquatic or airborne pathogens and microbial communities. Although few studies have characterized the conjunctival-associated lymphoid tissue (CALT) in the ocular mucosa (OM) of birds and mammals, little is known regarding the evolutionary origins and functions of immune defense and microbiota homeostasis of the OM in the early vertebrates.

**Results:**

Our study characterized the structure of the OM microbial ecosystem in rainbow trout *(Oncorhynchus mykiss*) and confirmed for the first time the presence of a diffuse mucosal-associated lymphoid tissue (MALT) in fish OM. Moreover, the microbial communities residing on the ocular mucosal surface contribute to shaping its immune environment. Interestingly, following IHNV infection, we observed robust immune responses, significant tissue damage, and microbial dysbiosis in the trout OM, particularly in the fornix conjunctiva (FC), which is characterized by the increase of pathobionts and a reduction of beneficial taxa in the relative abundance in OM. Critically, we identified a significant correlation between viral-induced immune responses and microbiome homeostasis in the OM, underscoring its key role in mucosal immunity and microbiota homeostasis.

**Conclusions:**

Our findings suggest that immune defense and microbiota homeostasis in OM occurred concurrently in early vertebrate species, shedding light on the coevolution between microbiota and mucosal immunity.

Video Abstract

**Supplementary Information:**

The online version contains supplementary material available at 10.1186/s40168-023-01716-6.

## Background

The eye is a highly specialized sensory organ that plays critical roles in finding food, attracting mates, and evading predators for the survival and reproduction of vertebrates [1]. The vertebrate-style eye, also known as the camera-like eye, first emerged approximately 500 million years ago (Mya) in lampreys, one of the most ancient jawless fish among living vertebrate species [2–4]. Interestingly, the cornea (Cor) of the lamprey may hold key insights into the early primordial development of vertebrate eyes, as it is merely an extension of the sclera covered with transparent skin [5]. Unlike jawless fish, sharks have immovable eyelids and a nictitating membrane, which serves different functions from those of tetrapods, primarily acting as a protective mechanism during feeding or when facing potential threats [6, 7]. Bony fishes lack true eyelids, and their eyeballs are separated from the surrounding skin by a shallow circumferential depression between the corneal epithelium (EP) and the skin [8]. When vertebrates first colonized terrestrial environments, their ocular surface underwent further evolutionary adaptations, including the development of novel adnexa such as the lacrimal gland, movable eyelids, and eyelashes in the case of mammals. These structures provide protection for the eye, keeping the Cor clean and moist, and enabling adaptation to dry land environments [9–12]. It is important to note that throughout the evolutionary timeline of vertebrates, the ocular surface underwent significant adaptive changes required for survival under various environmental pressures, particularly during the water-to-land transition [13].

The ocular mucosa (OM) in vertebrates is a layer of mucous membrane that lines the surface of the eyeball and eyelid, providing a physical and immunologic barrier against various challenges [14, 15]. Interestingly, the OM of most vertebrates presents a similar structure, mainly consisting of the Cor and conjunctiva (Figure S1). However, jawless fish such as lampreys only have a Cor [13]. The Cor of vertebrates is composed of two main layers: the EP and the stroma (ST), with the EP featuring a stratified, non-keratinized squamous layer [16]. Moreover, distinct populations of resident immune cells in the corneal EP, including innate lymphoid cells, Langerhans cells, mast cells, macrophages, and T cells, form a complex immune network that enables the Cor to mount prompt immune responses to different environmental challenges [17, 18]. The conjunctiva consists of an outer stratified EP richly interspersed with goblet cells, along with an underlying loose connective tissue known as the lamina propria (LP) [19]. In birds and mammals, lymphocytes reside on the conjunctival surface, forming a mucosa-associated lymphoid tissue (MALT) known as the conjunctiva-associated lymphoid tissue (CALT), which functions to detect antigens and contributes to the regulation of the local immune response Knop [19, 20]. Notably, although the Cor and the conjunctiva are anatomically close and face similar environmental challenges and stress, the immune defensive mechanisms in these tissues are distinctly different [17]. Critically, the Cor enjoys immune privilege, as its microenvironment is anti-inflammatory and immunosuppressive, thus ensuring the Cor’s transparency. On the other hand, the conjunctiva is a highly reactive tissue that can mount a potent immune response, which is important for clearing pathogens in the OM [17, 19].

Commensal microbiota and the immune system have coevolved in animals over the course of millions of years, and their interactions are dynamic and intertwined [21, 22]. It is now widely recognized that there is a bidirectional relationship between the microbiome and the immune system. Specifically, the microbiota plays a critical role in training and developing the host immune system, whereas the immune system regulates and shapes the microbiome in various mucosal tissues [22]. Similar to other mucosal surfaces, OM in mammals also hosts a unique microbial community. Previous studies have suggested that an imbalance in microbial homeostasis in the OM can lead to local or systemic inflammatory responses and eye diseases [23, 24]. Although it is known that the OM in mammals contains MALTs, which are essential for maintaining microbial homeostasis [17, 25], very little is known regarding the evolutionary origins of OM immunity in early vertebrate species and its primordial roles in immune defense and microbiota homeostasis. Teleost fish represent the oldest bony vertebrates and lack eyelids on their ocular surfaces. Their OM may face strong challenges from aquatic environments. Given the mucosal nature and similar evolutionary forces acting on the OM in vertebrates, we hypothesized that both primitive and modern bony vertebrates have evolved similar immune mechanisms to maintain microbial homeostasis in the OM.

Confirming our hypothesis, we present the first evidence that teleost fish OM possesses a well-defined MALT with structural and functional immune characteristics similar to those described in mammals and other fish species. Importantly, we demonstrate that the trout OM can mount robust antiviral immune and inflammatory responses upon viral infection, resulting in severe tissue damage to the OM epithelial layer. This damage leads to bacterial translocation and profound dysbiosis characterized by a loss of beneficial taxa and the proliferation of pathobionts, followed by a strong antibacterial response. Interestingly, correlation analysis revealed a positive correlation between most antibacterial and inflammatory genes with pathobionts, whereas a negative correlation was observed with beneficial bacteria. Furthermore, we observed a reversal of tissue damage and microbial translocation, as well as the restoration of microbiome homeostasis, accompanied by the disappearance of the inflammatory response. Overall, our findings uncover a previously unrecognized role of teleost fish OM in immune defense and maintaining microbial homeostasis, providing insights into the coevolution between microbiota and mucosal immunity in the OM that emerged in early vertebrate species.

## Results

### The teleost OM shares common anatomical features with other vertebrates

**Fig. 1:**
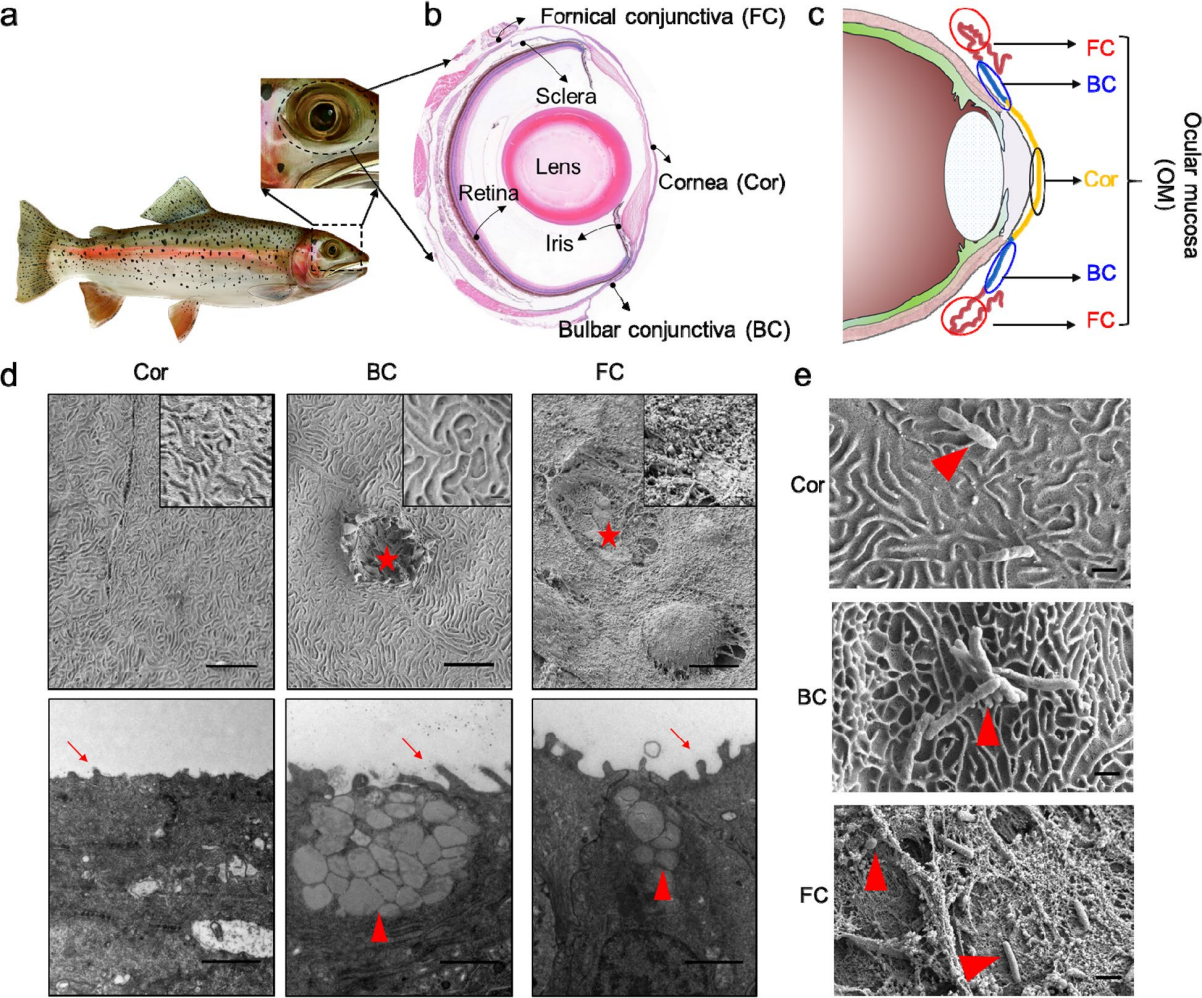
Structural characteristics in the Cor, BC, and FC of trout OM. **a** Trout and its eye enlargement. **b** H&E stain of trout eye. **c** Schematic diagram of the existence of three distinct regions on trout OM. d TEM (upper) and scanning electron microscope (lower) of the three distinct regions of control trout OM. From left to right: Cor, BC, and FC. Red stars indicate goblet cell orifices, red arrowheads indicate goblet cells, red arrows indicate microvilli. Scale bar, 2 μm. Enlarged images shown in (d, upper) refer to control trout OM. e Scanning Electron Microscope of microorganisms in three regions of the Cor (upper), BC (middle), and FC (lower) of control trout OM. Red arrowheads indicate commensal microbiota. Scale bar, 1 μm

The eyes are the most important sensory organs for fish, and in most fish species, the eyes are positioned on the sides of their heads without eyelids, as seen in tetrapods (Fig. 1a). To examine the basic structure of the teleost eye, we stained paraffin sections of trout eyes with hematoxylin and eosin (H&E) (Fig. 1b). Our findings reveal that the structure of teleost fish eyes is basically similar to that of tetrapods, including the lens, iris, sclera, retina, Cor, and conjunctiva (which can be further divided into bulbar conjunctiva (BC) and fornix conjunctiva (FC)) (Fig. 1b, c). Histological examination of the trout eyes showed that the ocular surface is covered by a mucous membrane known as the OM, primarily consisting of the Cor, BC, and FC (Fig. 1b, c). Next, we conducted histological analysis via H&E (Figure S2, upper) and Alcian Blue (AB) (Figure S2, lower) staining of OMs obtained from five different teleost families (Figure S3): Tetraodontidae, Centrarchidae, Salmonidae, Loricariidae, and Cyprinidae. The OMs of all species examined displayed a similar overall structure, including rainbow trout (*Oncorhynchus mykiss*) (Figure S2a), Japanese pufferfish (*Takifugu rubripes*) (Figure S2b), largemouth bass (*Micropterus salmoides*) (Figure S2c), common pleco (*Hypostomus plecostomus*) (Figure S2d), and common carp (*Cyprinus carpio*) (Figure S2e). As shown in Figure S2, the BC and FC exhibited two typical layers, namely the EP and LP, whereas the Cor consisted of the EP and ST. Additionally, AB staining revealed a large number of goblet cells in the EP of the BC and FC, whereas these cells were not observed in the EP of the Cor. Despite some minor morphological differences of five different teleost families, the OM all harbor myeloid and lymphoid cells mainly scattered in the EP of the BC and FC, similar to teleost MALTs found in the skin, gut, nose, and gills (Figure S2). Interestingly, using immunofluorescence microscopy, we observed that IgT^+^ and IgM^+^ B cells were present mostly within the epithelial layer (data not shown) and not the lamina propria unlike in the mammalian OM where they are predominantly dispersed in the follicles and lamina propria [25]. Transmission electron microscope (TEM) and scanning electron microscope images confirmed the AB staining results, showing the presence of goblet cells in the BC and FC, but not in the Cor. Furthermore, the OM displayed abundant microplicae and microvilli, with longer microvilli observed in the BC and FC compared to the Cor (Fig. 1d). These results highlight distinct differences in the organizational structure characteristics of the Cor, BC, and FC, suggesting the existence of different microenvironments or niches among them.

### Varying sites within the teleost OM harbor diverse and distinct commensal microbiota

**Fig. 2:**
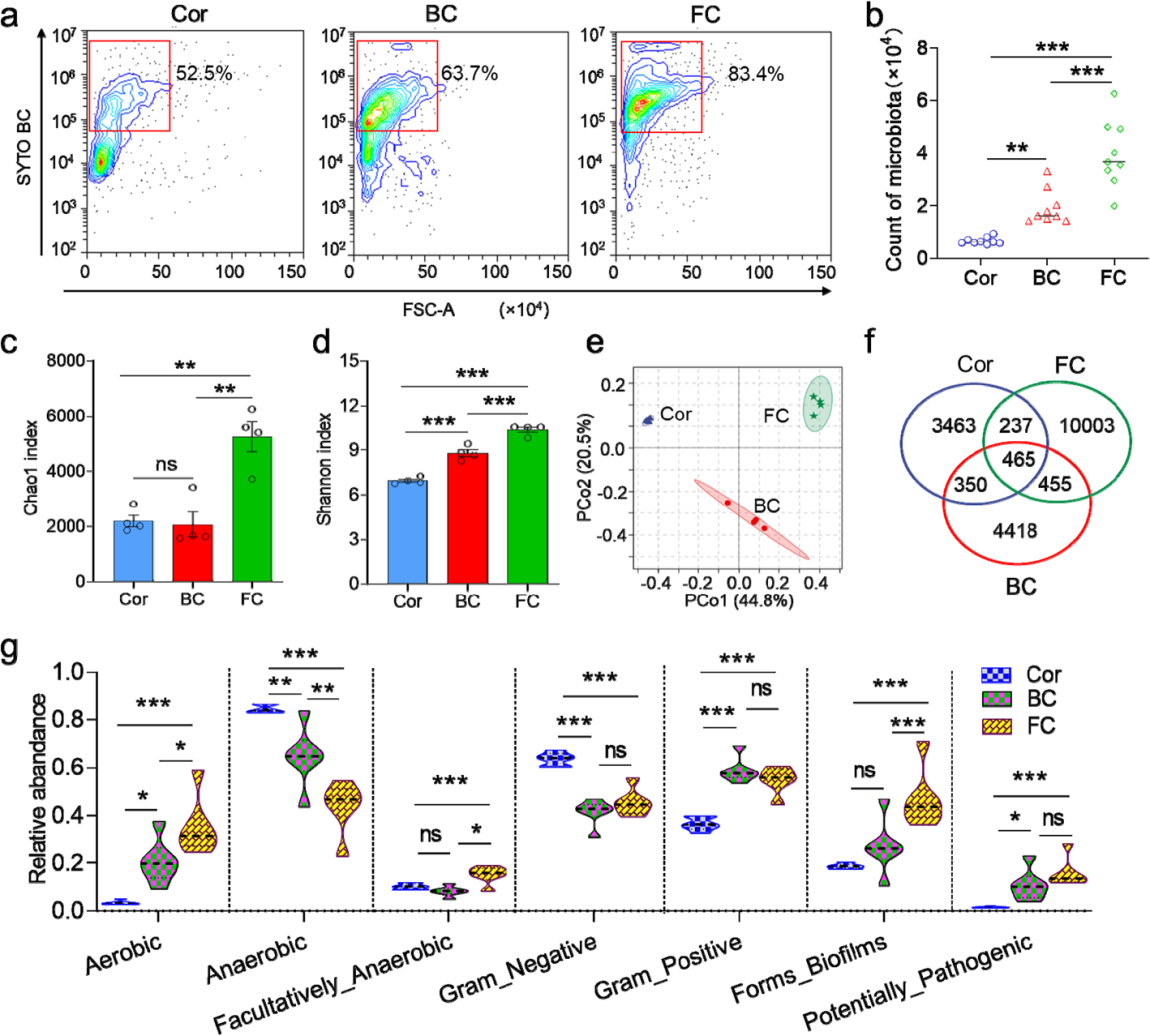
Distribution characteristics of commensal microbiota in the Cor, BC, and FC of trout OM. **a** Representative dot plots showing the staining of bacteria on the Cor, BC, and FC with SYTO BC Green. **b** Microbial counts on the Cor, BC, and FC (*n* = 9). **c**, **d** Richness (**c**) and diversity (**d**) of bacterial community in Cor, BC, and FC of control trout OM (*n* = 4). Community richness and diversity were measured by Chao1 index and Shannon index, respectively. e Principal coordinate analysis (PCoA) with weighted UniFrac distance matrix for the Cor, BC, and FC microbiota community from control trout OM (*n* = 4). **f** Venn diagram showing the number of the same and different bacteria in the Cor, BC, and FC of control trout OM. **g** Violin plot showing the representative abundance of aerobic-, anaerobic-, facultatively anaerobic-, gram-negative-, gram-positive-, forms biofilms-, and potentially pathogenic bacteria in the Cor, BC, and FC from control trout OM (*n* = 4). One-way ANOVA test was used to evaluate the statistical differences. Data are presented as mean ± SEM of three biological duplicates. *p < 0.05, **p < 0.01, ***p < 0.001

Teleost fish are known to have diverse microbial communities colonizing their mucosal surfaces [26–29]. Using scanning electron microscope, we observed the presence of bacteria in the Cor, BC, and FC (Fig. 1e), and the abundance of the ocular microbiome resembled that of the skin, but was lower than that of the gills and gut, as determined by qPCR analysis (Figure S4a). SYTO BC Green-Fluorescent Nucleic Acid staining was used to characterize the microbial distribution and composition in the Cor, BC, and FC. Here, we found that microbial abundance was highest in the FC, followed by the BC, and lowest in the Cor (Fig. 2a, b; samples without SYTO BC Green staining are shown in Figure S4b). Furthermore, analysis of 16S rRNA gene sequencing data confirmed the highest abundance of bacterial communities in the FC, with relatively lower abundances in the BC and Cor (Fig. 2c, d), as indicated by the Shannon and Chao1 diversity indices. These differences in the OM sites may be attributed to the Cor and BC being more exposed to water flushing, resulting in relatively less microbial colonization, whereas the FC, with its numerous folds, provides a favorable environment for microbial colonization. Principal coordinate analysis (PCoA) revealed distinct clustering patterns in the microbiome compositions of the Cor, BC, and FC (Fig. 2e). Venn diagram analysis also demonstrated marked differences in microbiota composition among the Cor, BC, and FC (Fig. 2f). Additionally, we observed lower abundance of aerobic and gram-positive bacteria in the Cor compared to the BC and FC, whereas anaerobic and gram-negative bacteria exhibited higher abundances in the Cor compared to the BC and FC. Importantly, the abundance of biofilm-forming and potentially pathogenic bacteria was higher in the FC compared to the Cor and BC (Fig. 2g), suggesting that the FC may experience stronger selection pressure from the microbial communities residing on its mucosal surface.

### Trout OM exhibits distinct immune characteristics in different anatomical niches

**Fig. 3:**
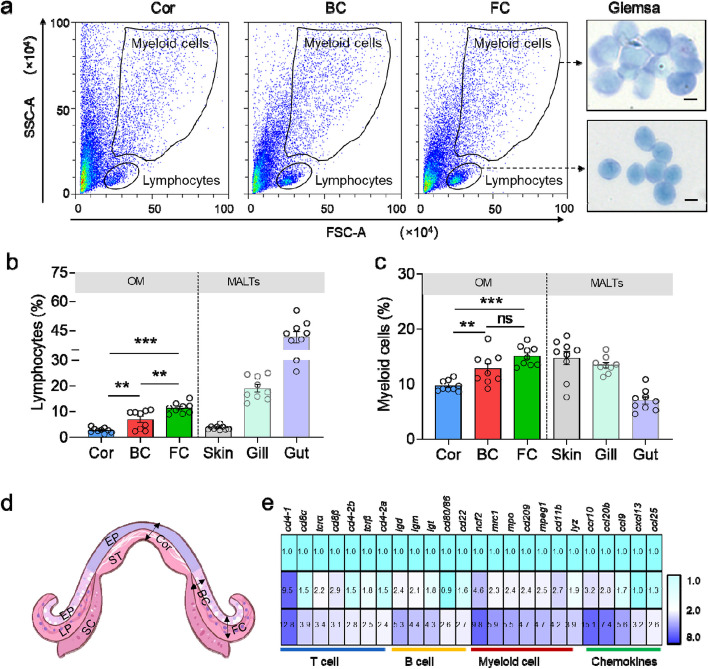
Trout OM exhibits distinct immune characteristics in different anatomical niches. a Dot plots showing lymphocytes and myeloid cells in the Cor, BC, and FC of control trout OM. Giemsa staining indicates the morphology of the sorted lymphocytes and myeloid cells (right). Scale bars, 5 μm. b Percentage of lymphocytes in the Cor, BC, FC, and other fish MALTs (i.e., skin, gill, and gut) of control trout (*n* = 9). **c** Percentage of myeloid cells in the Cor, BC, FC, skin, gill, and gut of control trout (*n* = 9). **d** Schematic diagram of the distribution of microdissection in the Cor, BC, and FC of control trout OM, EP, epithelium; ST, stroma; LP, lamina propria. **e** Heatmap plot of qPCR test of mRNA levels of the studied immune markers in the Cor, BC, and FC of control trout OM (*n* = 9). Expression levels in the BC and FC were normalized to those in the Cor, which is set as 1. One-way ANOVA test was used to evaluate the statistical differences. Data are presented as mean ± SEM of three biological duplicates. **p < 0.01, ***p < 0.001

To determine the presence of immune components in trout OM, we conducted flow cytometry analysis to characterize immune-related cells in the Cor, BC, and FC at steady state. Our findings revealed typical lymphocyte populations and myeloid cell populations in these OM regions (Fig. 3a), similar to other mucosal tissues such as the skin, gills, and gut (Figure S5). Interestingly, the percentage of lymphocytes in trout OM was comparable to that in the skin, but significantly lower than that in the gills and gut. Notably, the FC exhibited a higher proportion of lymphocytes and myeloid cells compared to the Cor and BC, suggesting a prominent immune role of the FC in trout OM (Fig. 3b, c). To further investigate the immune gene expression profile in different sites of trout OM, we dissected three regions using laser capture microdissection (LCM) (Fig. 3d) and extracted total RNA for immune gene expression analysis. Our results demonstrated that the FC had significantly higher expression levels of T cell-related markers (*cd4-1*, *cd8α*, *tcrα*, *cd8β*, *cd4-2b*, *tcrβ*, and *cd4-2a*), B cell-related markers (*igd*, *igm*, *igt*, *cd80/86*, and *cd22*), myeloid cell-related markers (*ncf2*, *cd11b, mrc1*, *mpeg1*, *mpo*, *cd209*, and *lyz*), as well as cytokines (*ccr10*, *ccl20b*, *ccl9*, *cxcl13*, and *ccl25*) compared to the Cor and BC (Fig. 3e). Collectively, these results indicate that trout OM constitutes a unique immune cell microenvironment and exhibits a distinct immune transcriptional profile under a steady state.

### The microbiome contributes to shaping the teleostocular immune environment

**Fig. 4:**
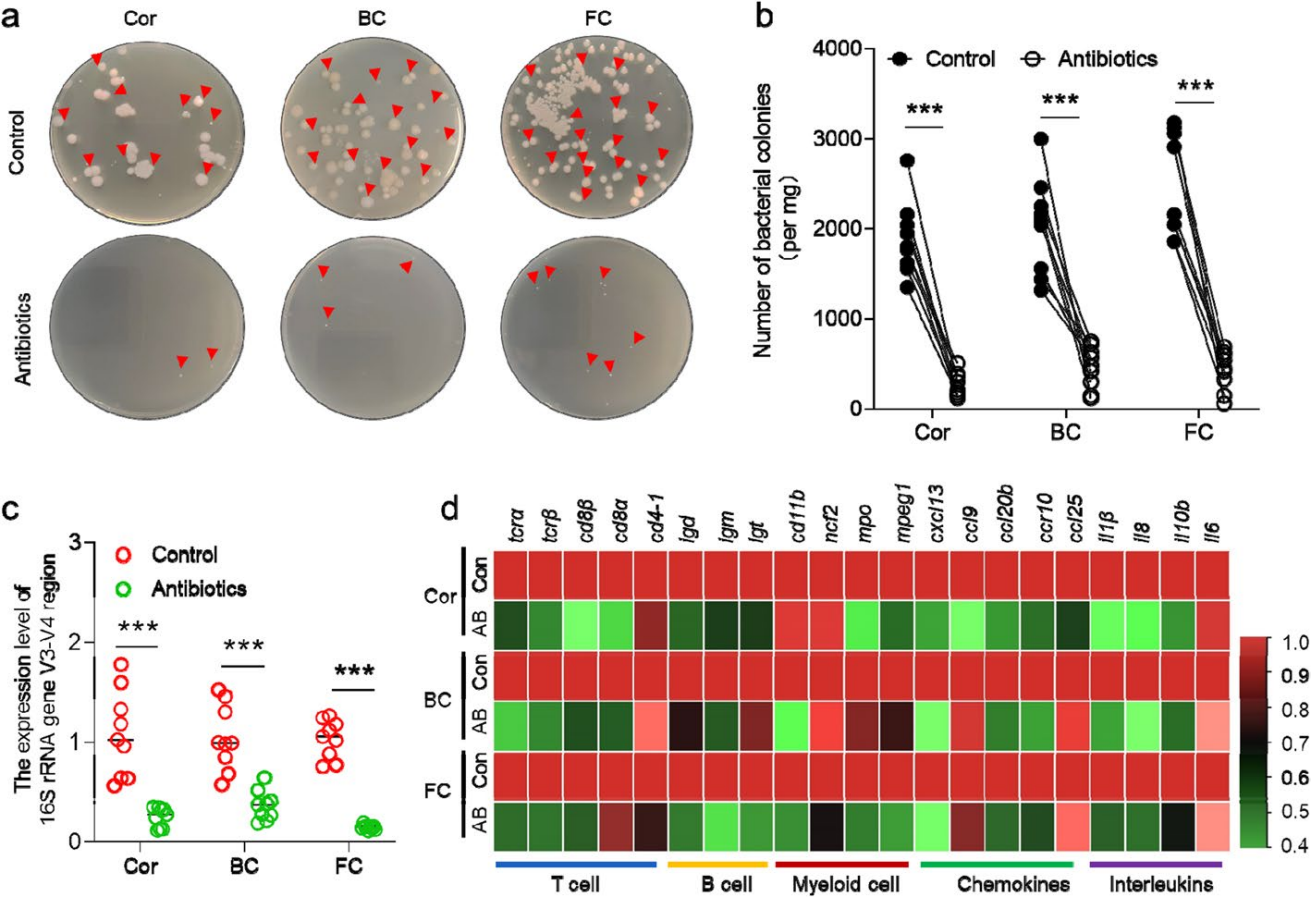
Effects of antibiotics on OM commensal bacteria and immunity. **a** The culture plates from trout OM (from left to right: Cor, BC, and FC) of control fish (upper) and 4-day treated fish with antibiotics (lower), respectively. Red triangles indicate a single colony of bacteria. **b** Numbers of bacterial colony in control and 4-day treated fish counted from a (*n* = 9). **c** The expression of V3-V4 16S rRNA region in the Cor, BC, and FC after treatment with antibiotics. **d** The expression of immune-related genes in the trout Cor, BC, and FC after treatment with antibiotics. AB, antibiotics. Statistical differences were evaluated by unpaired Student’s *t* test. ***p < 0.001

To further evaluate whether the high abundance of microbiota residing on the ocular mucosal surface contributes to shaping its immune environment, we measured the expression level of immune-related genes in the trout OM after exposure to a mixture of antibiotics. After 4 days, we performed an initial analysis of the abundance of microbiota using plate count and qPCR (Fig. 4). Our results indicated that the number of colonies and the expression levels of 16 s rRNA V3-V4 region decreased markedly in Cor, BC, and FC tissues treated with the antibiotics compared to the control group (Fig. 4a–c). Subsequently, we detected the expression of T cell-related markers (*tcrα*, *tcrβ*, *cd8α*, *d8β*, *cd4-1*,), B cell-related markers (*igd*, *igm*, *igt*), myeloid cell-related markers (*ncf2*, *cd11b, mpeg1*, and *mpo*), chemokines (*ccr10*, *ccl20b*, *ccl9*, *cxcl13*, and *ccl25*), as well as interleukins (*il1β*, *il8*, *il10b*, and *il6*) in the OM and found the expression of these immune-related genes also decreased significantly in the antibiotics treatment group (Fig. 4d). Taken together, these findings suggest that microbiota in OM plays a crucial role in shaping the ocular immune environment.

### The OM of trout mounts a robust immune response upon IHNV infection

**Fig. 5:**
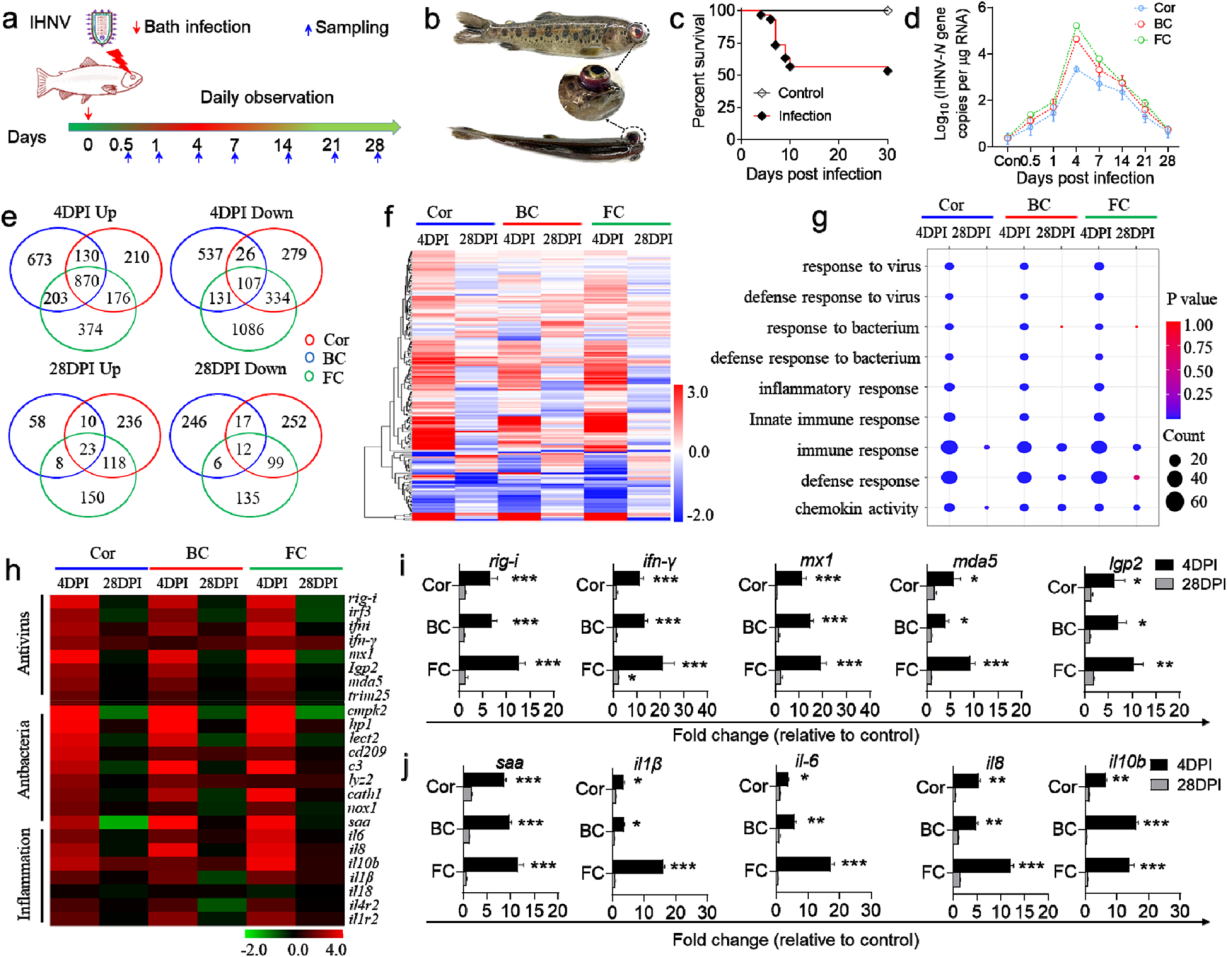
Infection model and transcriptome analyses of trout OM with IHNV. **a** Scheme of the experimental strategy. Fish were infected by bathing with IHNV, and subsequently sacrificed at 0.5, 1, 4, 7, 14, 21, and 28 DPI for tissue sample collection. **b** Clinical signs of trout infected by IHNV at 4 DPI with exophthalmia and petechial hemorrhages. **c** Cumulative survival of IHNV-infected fish and control group. Statistical differences were assessed by the Log-rank (Mantel-Cox) test. **d** qPCR was applied to detect IHNV-*N* gene copies (Log_10_) in the trout Cor, BC, and FC collected at 0.5, 1, 4, 7, 14, 21, and 28 DPI (*n* = 6). **e** Venn diagrams showing the overlap of up- or downregulated genes detected by RNA-seq in the trout Cor, BC, and FC at 4 or 28 DPI versus control fish. **f** Heatmap plot of RNA-Seq studies for changed immune genes from the Cor, BC, and FC of IHNV-infected versus control fish measured at 4 and 28 DPI. **g** RNA-Seq data shows altered biological processes in the trout Cor, BC, and FC at 4 and 28 DPI versus control trout. **h** Heatmap plot of RNA-Seq studies for changed antivirus, anti-bacteria, and inflammation genes in the trout Cor, BC, and FC at 4 and 28 DPI. **i**, **j** Representative antiviral response (**i**) and inflammatory response (**j**) genes in the Cor, BC, and FC captured by LCM for control and IHNV-infected trout, and measured by qPCR. Gene expression levels in IHNV-infected fish were normalized to those in the control group, which were set as 1 (*n* = 9). Statistical differences were evaluated by unpaired Student’s *t* test. Data are presented as mean ± SEM of three biological duplicates. *p < 0.05, **p < 0.01, ***p < 0.001

We next evaluated whether the Cor, BC, and FC serve as immune sites and generate immune responses, we developed a bath infection model using IHNV and collected tissue samples at several time points (Fig. 5a). Our result showed that infected fish displayed typical symptoms such as exophthalmia and petechial hemorrhages around the eyes at 4 DPI (Fig. 5b), and approximately 44% of the fish died within 2 weeks of infection (Fig. 5c). Furthermore, the FC exhibited a higher viral load after IHNV infection compared to the Cor and BC. The viral load in all three sites peaked at 4 DPI and gradually decreased, reaching pre-challenge levels at 28 DPI (Fig. 5d). Similar results were observed in OM paraffin sections stained with an anti-IHNV-*N* monoclonal antibody (Figure S6a, b; isotype-matched control Abs as shown in Figure S7). Consistently, H&E staining further confirmed these findings (Figure S6c, d). This suggests that the FC may be one of the primary targets of the virus. Taken together, our results confirm the successful establishment of an IHNV infection model in the OM of trout.

Next, to assess the immune responses of trout OM, we conducted transcriptome sequencing analyses of the Cor, BC, and FC at 4 and 28 DPI. RNA-Seq analysis revealed significant modifications in a total of 2132 genes (Cor), 2677 genes (BC), and 3,281 genes (FC) at 4 DPI following IHNV infection compared to the controls. Among these genes, 1386, 1876, and 1623 were upregulated, whereas 746, 801, and 1,658 were downregulated in the Cor, BC, and FC, respectively. Similarly, at 28 DPI following IHNV infection, a total of 767 genes (Cor), 380 genes (BC), and 551 genes (FC) were significantly modified, with 378, 99, and 299 genes being upregulated, and 380, 281, and 252 genes being downregulated in the Cor, BC, and FC, respectively (Fig. 5e). Furthermore, we observed a significant upregulation of numerous immune-related genes in the Cor, BC, and FC at 4 DPI, with expression levels returning to nearly normal levels at 28 DPI (Fig. 5f). These differentially expressed genes (DEGs) were primarily enriched in gene pathways related to antiviral, antibacterial, and inflammatory responses (Fig. 5g), as well as pathways associated with pattern recognition receptors, B and T cell receptors, and chemokine signaling (Figure S6) at 4 DPI. Specifically, at 4 DPI, several antiviral genes (*rig-i*, *irf3*, *ifni*, *ifn-γ*, *mx1*, *lgp2*, *mda5*, and *trim25*), antibacterial genes (*cmpk2*, *hp1*, *lect2*, *cd209*, *c3*, *lyz2*, *cath1*, and *nox1*), and inflammation genes (*saa*, *il6*, *il8*, *il10b*, *il1β*, *il18*, *il4r2*, and *il1r2*) were significantly upregulated (Fig. 5h). At 28 DPI, the expression of these genes had largely returned to the pre-challenge levels (Fig. 5g, h, Figure S8). To further explore the immune responses of trout Cor, BC, and FC, we dissected the three regions using LCM and extracted total RNA to detect changes in immune genes. Consistent with the transcriptome results, qPCR analysis demonstrated a strong immune response in the Cor, BC, and FC at 4 DPI, as indicated by a significant increase in the expression of antiviral (*rig-i*, *ifn-γ*, *mx1*, *mda5*, and *lgp2*) and inflammatory genes (*saa*, *il1β*, *il6*, *il8*, and *il10b*). The immune response gradually returned to homeostasis at 28 DPI (Fig. 5i, j). Notably, the FC of infected trout exhibited a stronger immune response, both antiviral and inflammatory, compared to the Cor and BC (Fig. 5i, j). These findings suggest that IHNV infection elicits a stronger immune response in the trout FC, highlighting its significant role in the antiviral infection within the local OM environment.

### IHNV infection causes bacterial translocation in trout OM

**Fig. 6:**
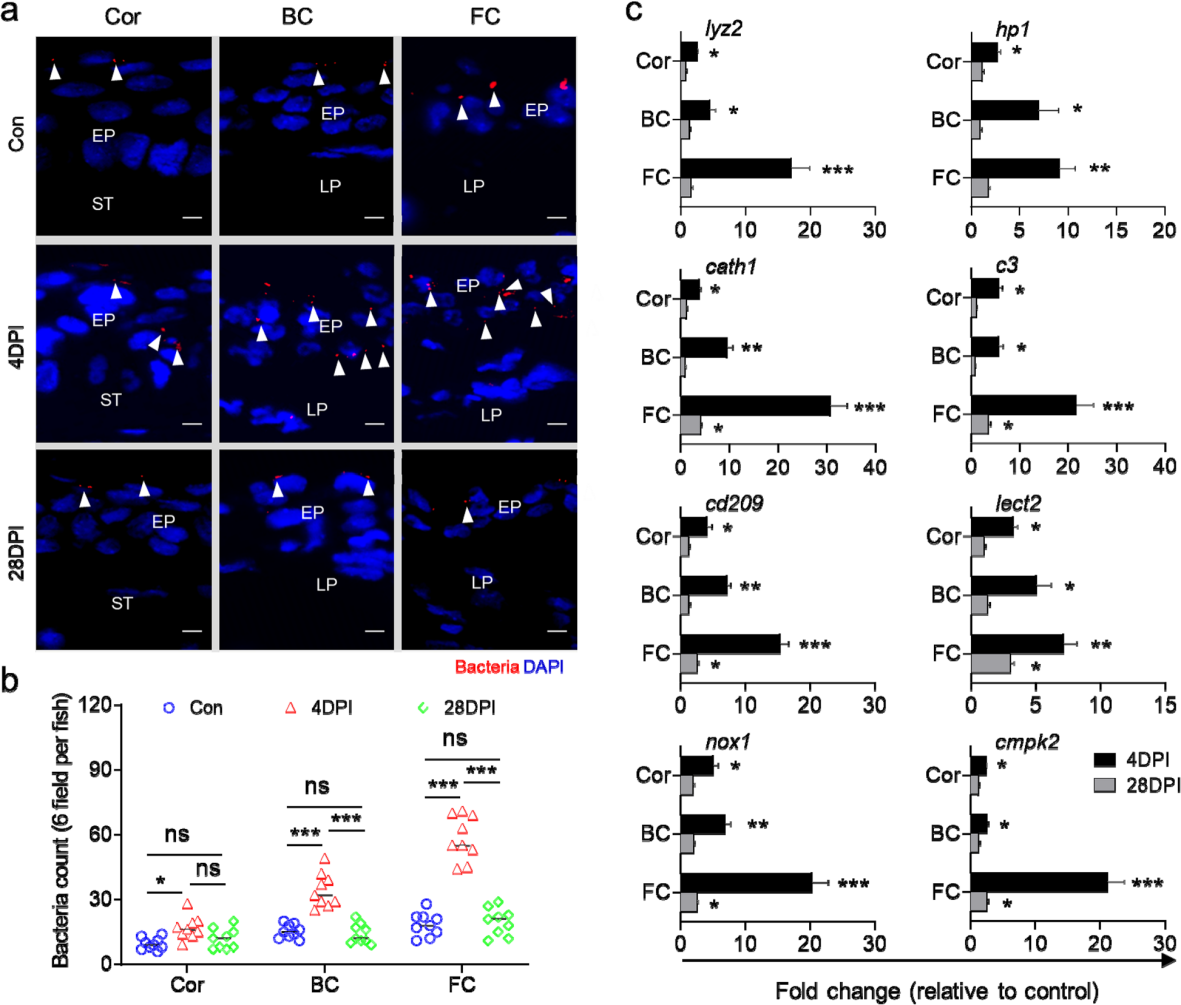
IHNV infection leads to microbiota translocation and subsequently induces an antibacterial immune response. **a** Fluorescence in situ hybridization experiment to detect the bacteria in the Cor, BC, and FC paraffin-sections from 4 and 28 days infected and control trout. From left to right: Cor, BC, and FC. EP, epithelium; ST, stroma; LP, lamina propria. Bacteria were stained red with Cy5-EUB338 oligoprobe, while nuclei were stained blue with DAPI. White arrowheads indicate bacteria. Scale bars, 10 μm. **b** Counts of positive bacteria in the Cor, BC, and FC of the control group and IHNV-infected trout at 4 and 28 DPI (*n* = 9). One-way ANOVA test was used to evaluate the statistical differences. **c** qPCR analysis of anti-bacteria genes in the Cor, BC, and FC of control and IHNV-infected trout OM at 4 and 28 DPI. Gene expression levels in IHNV-infected trout were normalized to those in the control group, which were set as 1 (*n* = 9). Statistical differences were evaluated by unpaired Student’s *t* test. Data are presented as mean ± SEM of three biological duplicates. *p < 0.05, **p < 0.01, ***p < 0.001

The tissue damage and antibacterial immune response triggered by IHNV infection led us to speculate whether it could disrupt microbial homeostasis. Through fluorescent in situ hybridization (FISH) analysis, we observed a significant translocation of bacteria from the mucus layer across the EP in the Cor, BC, and FC at 4 DPI compared to the control group. The number of translocated bacteria was higher in the FC, but this was substantially restored at 28 DPI (Fig. 6a, b). To further investigate whether bacterial translocation into the mucosal EP induced an antibacterial immune response in the trout OM, we analyzed the transcript levels of antibacterial genes (*lyz2*, *hp1*, *cath1*, *c3*, *cd209*, *lect2*, *nox1*, and *cmpk2*) in the Cor, BC, and FC using LCM. We observed a significant increase in these genes at 4 DPI (Fig. 6c). Consistent with the histopathological changes and inflammatory response, a stronger antibacterial immune response was observed in the FC compared to the Cor and BC (Fig. 6c). Overall, our findings demonstrate that IHNV infection may lead to secondary bacterial infection, resulting in an antibacterial immune response in trout OM.

### IHNV infection induces profound dysbiosis in the OM of trout

**Fig. 7:**
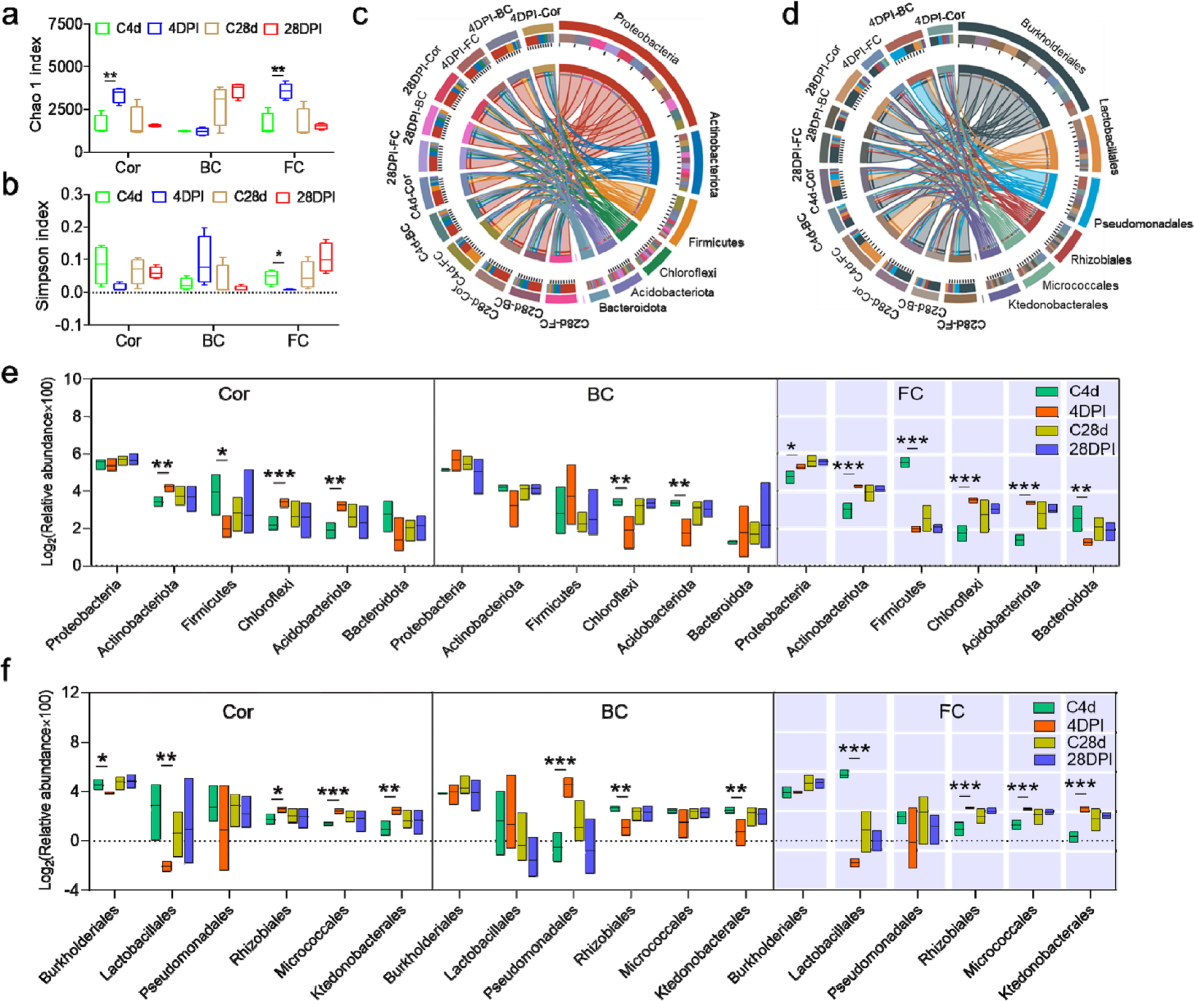
IHNV infection leads to dysbiosis of trout OM microbiota. a, b Richness and diversity of bacterial community in the Cor, BC, and FC of control and IHNV-infected fish at 4 and 28 DPI (*n* = 4), measured with Chao1 and Simpson index, respectively. **c**, **d** Circos plots display the corresponding abundance of samples in relation to bacterial communities at the phylum (**c**) and order (**d**) levels. e Relative abundance (%) of the top six bacteria (phylum) in the Cor, BC, and FC. f Relative abundance (%) of the top six bacteria (order) in the Cor, BC, and FC. Statistical differences were assessed by unpaired Student’s *t* test. Data are presented as mean ± SEM of three biological duplicates. *p < 0.05, **p < 0.01, ***p < 0.001

To investigate the effect of IHNV infection on the microbial composition of the OM, trout Cor, BC, and FC were collected from control and IHNV-infected trout at 4 and 28 DPI for 16S rRNA sequencing analysis. The analysis of Chao 1 indices revealed a significant increase in the richness of bacterial communities in the Cor and FC at 4 DPI, whereas it remained almost unchanged in the BC (Fig. 7a). Additionally, the diversity of bacterial communities in the FC was significantly decreased at 4 DPI, but not in the Cor and BC (Fig. 7b). A circos plot was generated to visualize the changes in bacterial structure at the phylum and order levels in OM between control and infected fish (Fig. 7c, d). At the phylum level, we observed a significant increase in the abundance of Actinobacteriota, Chloroflexi, and Acidobacteriota, and a decrease in Firmicutes in the Cor at 4 DPI. In the BC, Chloroflexi and Acidobacteriota were significantly decreased. Conversely, significant changes occurred in all top six phyla in the FC. Specifically, Proteobacteria, Actinobacteriota, Chloroflexi, and Acidobacteriota increased significantly, whereas Firmicutes and Bacteroidota decreased significantly at 4 DPI (Fig. 7e). At the order level, IHNV infection resulted in a significant increase in the abundance of Rhizobiales, Micrococcales, and Ktedonobacterale, and a significant reduction in Lactobacillales and Burkholderiales in the Cor. In the BC, there was a significant decrease in the abundance of Rhizobiales and Ktedonobacterale, coupled with an increase in Pseudomonadaies at 4 DPI. Interestingly, similar changes were observed in the FC tissue at the order level, with an increase in Rhizobiales, Micrococcales, and Ktedonobacterale, and a decrease in Lactobacillales at 4 DPI (Fig. 7f).

**Fig. 8:**
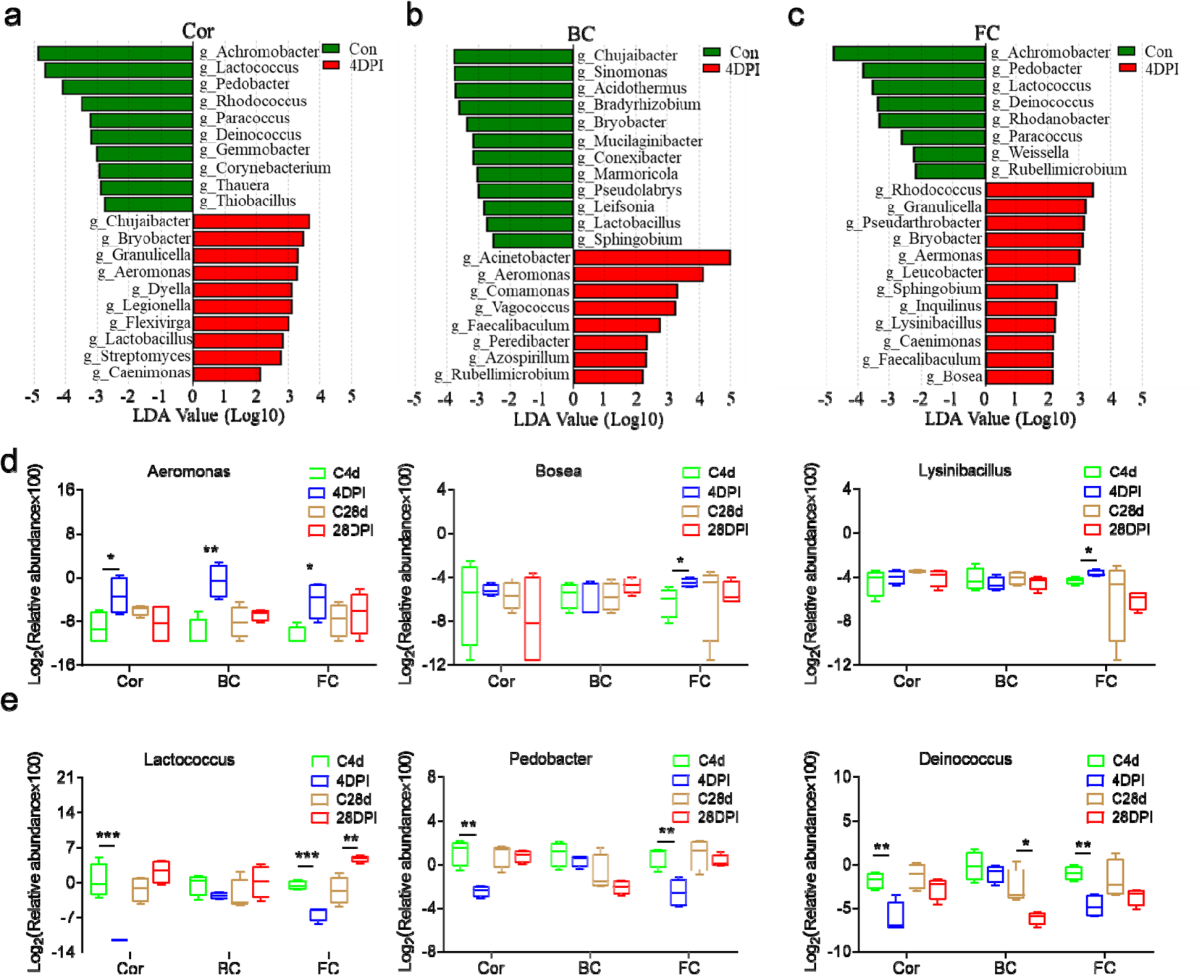
Differences of bacterial taxa in Cor, BC and FC after IHNV infection. **a**–**c** Bar chart of the log-transformed LDA value of bacterial taxa found to be significantly associated with control fish and trout infected with IHNV at 4 DPI in Cor (**a**), BC (**b**), and FC (**c**) by LEfSe (*p* < 0.05). **d** Relative abundance of the pathogenic bacteria (*Aeromonas*, *Bosea*, and *Lysinibacillus*) in the Cor, BC, and FC. **e** Relative abundance of the beneficial bacteria (*Lactococcus*, *Pedobacter*, and *Deinococcus*) in the Cor, BC, and FC. Statistical differences were assessed by unpaired Student’s *t* test. Data are presented as mean ± SEM of three biological duplicates. *p < 0.05, **p < 0.01, ***p < 0.001

LEfSe (linear discriminant analysis effect size) analysis was conducted next to further explore the microbial biomarkers contributing to the structural changes in bacteria among the different sites (Fig. 8a–c, Figure S9a–c). Significant changes in bacterial composition were observed in the Cor, BC, and FC tissues at 4 DPI (Fig. 8a–c). Specifically, there was a significant increase in *Aeromonas* abundance in all three regions, whereas the abundance of *Bosea* and *Lysinibacillus* increased significantly in the FC at 4 DPI (Fig. 8d). Furthermore, *Deinococcus* abundance decreased significantly in all of the examined regions of trout OM, whereas *Lactococcus* and *Pedobacter* only decreased significantly in the Cor and FC at 4 DPI. Interestingly, the abundance of *Lactococcus* significantly increased at 28 DPI (Fig. 8e), possibly indicating a return to homeostasis as the dominant bacterial group. Importantly, as the inflammatory response dissipated at 28 DPI, the bacterial community composition of the trout OM exhibited minor differences compared to the control fish (Figs. 7 and 8d, e, Figure S9a–c), suggesting a strong association between microbial imbalance in trout OM and the inflammatory response.

### Analysis of the correlations between the relative abundance of the disordered microbiome and immune-related genes in the OM of trout

**Fig. 9:**
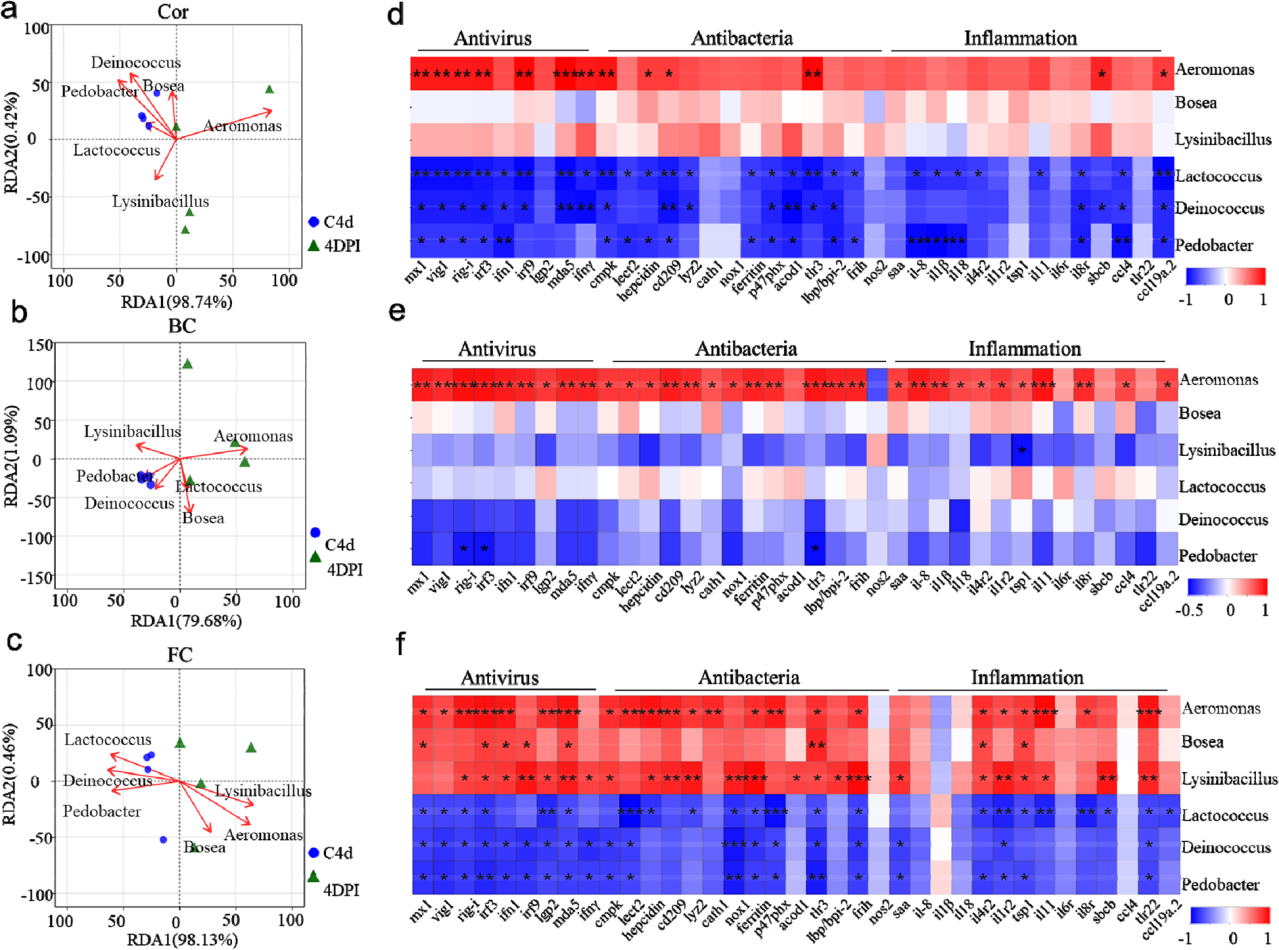
Correlation analysis between the relative abundance of the disordered microbiota and immune-related genes. **a**–**c** RDA association analysis of the Cor (**a**), BC (**b**), and FC (**c**) microbial genera and immune-related genes. **d**–**f** Spearman’s correlation between 37 immune-related genes expression determined by RNA-Seq and microbiota at genus level in the Cor (**d**), BC (**e**), and FC (**f**). Color scale shows a negative (blue) to positive (red) correlation. *p < 0.05, **p < 0.01, ***p < 0.001 indicated significant correlation

The large number of microorganisms inhabiting mucosal surfaces is closely linked to immunity [30]. In this study, we conducted correlation analyses between the relative abundance of OM microbiota at the genus level and immune-related genes, specifically inflammation, antiviral, and antibacterial genes, using distance-based redundancy analysis (db-RDA) (Fig. 9a–c) and Spearman correlation analysis (Fig. 9d–f). Our findings revealed a strong association between OM microbiota and immune-related genes. Notably, changes in *Aeromonas* abundance in the three regions of trout OM were positively associated with the levels of antibacterial and inflammation genes at 4 DPI (Fig. 9a–f), suggesting that *Aeromonas* may be the primary contributor to the dramatic inflammatory, antiviral, and antibacterial responses in the OM. Furthermore, variations in the relative abundance of pathogenic bacteria (*Bosea* and *Lysinibacillus*) were only positively related to most immune-related genes in the FC. *Bosea* was significantly associated with the antiviral gene (*mx1*, *irf3*, *irf9*, *ifn1*, and *mad5*), antibacterial gene *tlr3* and inflammation genes *il4r2* and *tsp1* (Fig. 9c, f). Importantly, we found that beneficial bacteria such as *Lactococcus*, *Deinococcus*, and *Pedobacter* were negatively associated with most antiviral, antibacterial, and inflammation genes (Fig. 9a–f). This suggests that pathogenic bacteria may largely contribute to the expression of antiviral, antibacterial, and inflammation genes, whereas beneficial bacteria play the opposite role. Interestingly, most immune-related genes significantly associated with beneficial bacteria were observed in the Cor and FC, but not in the BC (Fig. 9d–f). These observations suggest that IHNV infection may impose various selective pressures on the microbiota of different anatomical niches, leading to unique variations in the OM microbiome. Furthermore, these data indicate that there is a cross-talk between microbiota and immunity in the trout OM.

## Discussion

The eye is a functionally conserved sensory organ among vertebrates and has been subjected to similar selective forces from pathogens and mucosal microbial communities, regardless of whether the organisms are aquatic or terrestrial. Therefore, it is likely that they have evolved similar strategies to control pathogens and maintain ocular microbiota homeostasis. However, the immune roles of OMs have mainly been studied in mammals and birds, whereas little is known regarding the primordial roles of teleost OM in mucosal homeostasis. In this study, we provide the first evidence of a well-defined MALT in teleost OM and reveal its previously unrecognized role in immune defense and maintaining microbial homeostasis. This provides evolutionary insights into the potential interaction between microbiota and mucosal immunity in the OM of early vertebrates.

Here, we found that the anatomical structure of teleost OM resembles that of other jawed vertebrates, primarily consisting of the Cor and conjunctiva [31]. Since teleost fish lack eyelids, the conjunctiva can be further divided into two regions: the BC and FC, unlike sharks and tetrapods [6, 12], which have palpebral conjunctiva. Additionally, similar to other teleost MALTs [32], we observed the presence of a diverse microbial community in trout OM, although its abundance was significantly lower than that of the skin, gills, and gut. Our results support the notion that the diversity and abundance of bacteria are often influenced by the specific anatomical niche in which they reside [33]. Consistent with previous findings in the mammalian OM, we observed distinct bacterial communities in different OM sites (Cor, BC, and FC), with the FC exhibiting higher microbial richness and diversity. Moreover, in terms of phenotypes such as biofilm formation and potential pathogenicity, the relative abundance of microbiota in the FC was significantly higher than in the Cor and BC, indicating that FC is subject to greater selective pressure from the microbial communities residing on its mucosal surface. Another conserved feature of teleost MALTs, similar to mammalian MALTs, is the presence of numerous leukocytes (i.e., lymphocytes and myeloid cells) [34]. Here, we demonstrate for the first time that the teleost OM contains lymphocytes, myeloid cells, and cytokine expression, which is consistent with our previous findings in the teleost skin, gills, and gut [26–28]. Interestingly, we observed a higher abundance of leukocytes in the FC compared to the Cor and BC, suggesting a prominent role of the FC in trout OM immunity. Importantly, the abundance of lymphocytes at different locations strongly correlates with microbiota burden, thus highlighting the coevolutionary principles between microbiota and mucosal immunity in early vertebrate species. In mammals, the presence of microbes plays an essential role in shaping a favorable immune environment in the mucosa [35]. Studies have shown that the use of antibiotics leads to reductions in commensal microbes and alterations in microbiota composition, which in turn can impact the innate immune defense of the host [35–37]. In this study, we eliminated symbiotic microbiota with antibiotics treatment and found immune-related genes were significantly reduced in the Cor, BC, and FC at OM, which is consistent with previous findings in the mice [35], implying that microbiota is crucial in shaping the immune environment of the OM.

To further assess the immune responsiveness of the teleost OM, we established an IHNV infection model in trout through bath exposure, which mimics natural infection. As expected, the infected fish exhibited typical symptoms of IHN, including exophthalmia, petechial hemorrhages around the eyes, and darkening of the skin [38]. Furthermore, we observed a significant increase in IHNV load in the Cor, BC, and FC as early as 4 DPI, reaching near pre-challenge levels at 28 DPI. Notably, the FC showed the highest number of viral copies, whereas the Cor had very few viral copies, which was consistent with previous studies on human OM infection with SARS-CoV-2 [39]. Importantly, these findings correlate with severe histological changes in the EP of FC, suggesting that the FC is likely the primary entry point for this virus to invade the trout eye. Additionally, transcriptome analysis revealed the immune role of trout OM and unveiled its previously unrecognized capacity to respond to viral infection. Specifically, we observed an upregulation of antiviral genes, including *rig-i*, *mx1*, *ifn-γ*, *irf3*, and *mda5*, which are known to be crucial for effective antiviral responses in mucosal surfaces, particularly during the early stages of infection [40, 41]. Moreover, the IHNV challenge led to significant upregulation of pro-inflammatory cytokines (*il1β*, *il6*, and *il8*), anti-inflammatory cytokines (*il10b*), and inflammation regulation cytokines (*saa*, *il4r2*, and *il1r2*) at 4 DPI, with levels returning to baseline conditions at 28 DPI. Notably, consistent with findings in mammals that the conjunctiva is a highly reactive tissue and mounts a potent immune response to external stimuli [42, 43], we observed a stronger immune response in the trout conjunctiva compared to the Cor. Interestingly, within the conjunctiva, the FC exhibited a higher level of immune response than the BC, suggesting that the FC plays a pivotal role as an important effector site in teleost ocular mucosal immunity.

In mammals, previous studies have demonstrated that the inflammatory response triggered by a primary viral infection can increase susceptibility to secondary bacterial infections and disrupt the microbial balance in the conjunctiva [44, 45]. Consistent with this finding, we observed a significant increase in the levels of bacteria translocated from the mucus layer across the OM EP upon IHNV infection, particularly in the FC. Importantly, these findings correlated with the upregulation of antibacterial genes (*lyz2*, *hp1*, *cath1*, *c3*, *cd209*, *lect2*, *nox1*, and *cmpk2*), suggesting that IHNV infection may lead to microbiota translocation and subsequent strong antibacterial response. Furthermore, 16S rRNA analysis revealed a significant increase in microbial richness in the Cor and FC at 4 DPI. Notably, we observed a decrease in OM microbial diversity in the infected trout FC, as indicated by the Simpson index, which aligns with previous studies in mammals linking microbial diversity to various disorders [46, 47]. Additionally, similar to the ocular microbiome of humans, our results demonstrated that the trout OM harbors a diverse array of bacterial taxa belonging to six different phyla, representing approximately 90% of all OTUs. The dominant phyla in the teleost ocular microbiome were Proteobacteria (the most abundant phylum in human ocular microbiomes), followed by Actinobacteria and Firmicutes. Interestingly, the two dominant phyla in the trout ocular microbiome, Chloroflexi, and Acidobacteriota, are widely distributed in soil and freshwater sediments, whereas their abundance in the human ocular microbiome is low [48, 49]. Remarkably, the abundance of the Proteobacteria phylum in the trout FC microbial community significantly increased after IHNV infection. Proteobacteria are known to include various pathogens with adherent and invasive properties that can subvert host defenses and induce pro-inflammatory responses [50–52]. This finding is consistent with previous observations in mammalian conjunctiva [18]. Furthermore, we observed a significant decrease in the Firmicutes. This contributed to a reduced Firmicutes/Bacteroidetes ratio, which is known to be associated with dysbiosis and inflammatory diseases [53]. Therefore, our study demonstrates that while the composition and structure of the ocular microbiome in bony fish differ from those in mammals, the response of the ocular microbiota to pathogens is conserved across vertebrates.

Previous studies have reported that viral infections can disrupt the microbial balance and lead to ocular inflammation due to the proliferation of opportunistic pathogens [54, 55]. As expected, our findings show that IHNV infection in the trout OM resulted in an increase in pathogenic bacteria abundances, including *Aeromonas*, *Lysinibacillus*, and *Bosea*, which have been associated with ocular diseases in mammals [50, 56, 57]. Among them, *Aeromonas* species, which are widely distributed in natural environments, can trigger proinflammatory responses in a variety of hosts, including humans, aquatic animals, livestock, and poultry, through the release of various pro-inflammatory cytokines (e.g., *il-1β*, *il-6*, *il-8*, *il-12*, and *tnf-α*) [58, 59]. In addition, studies in mammals have reported that *Aeromonas* is capable of causing ocular infection leading to inflammation and abscesses [60, 61]. Notably, several antiviral-related genes (*irf3*, *mda5*, and *rig-i*) have been identified as key players in antimicrobial immune responses and the maintenance of microbial homeostasis [62–64]. Therefore, *Aeromonas* may contribute significantly to the proinflammatory response observed in trout OM at 4 DPI after IHNV infection. Interestingly, the significant increase in *Lysinibacillus* and *Bosea* was mainly observed in the FC, which are known to exacerbate inflammatory responses in the OM [57, 65]. Spearman’s correlation analysis further confirmed the positive correlation between the abundance of these pathogenic bacteria and the expression of antibacterial and inflammatory genes in the FC. These findings provide evidence that viral-induced microbial dysbiosis can lead to opportunistic pathogen expansion and reduction of beneficial commensals, ultimately resulting in ocular inflammation. Moreover, we observed a significant decrease in beneficial bacteria, such as *Lactococcus*, *Pedobacter*, and *Deinococcus*, primarily in the Cor and FC at 4 DPI. Importantly, most antiviral, antibacterial, and inflammatory genes showed a negative correlation with the abundance of *Lactococcus*, *Deinococcus*, and *Pedobacter*. These genera have been shown to reduce the levels of inflammatory cytokines (e.g., *tnf-α*, *il-1β*, *il-6*, and *il-8*) and play a crucial role in maintaining microbial homeostasis in the skin and gut [51, 66–68]. Recent studies have highlighted the potential of probiotic administration as a therapeutic approach for the treatment of ocular inflammatory diseases to regulate microbial homeostasis [69, 70]. However, most of our understanding of the relationship between symbiotic microbiota and OM immunity derives from studies in mammals. Therefore, to the best of our knowledge, our findings provide the first evidence of potential cross-talk between microbiota and immunity at the OM surface in an early vertebrate species.

## Conclusions

Our findings reveal the presence of a well-defined MALT in teleost OM and highlight the conserved principles shared by primitive and modern vertebrates in protecting mucosal sites from pathogens and maintaining microbiota homeostasis. The teleost OM exhibits a robust antiviral immune and inflammatory response upon viral infection, which is accompanied by tissue damage and bacterial translocation. Concurrently, viral-induced inflammatory responses lead to profound dysbiosis in the microbiome, which is characterized by the increase of pathobionts and a reduction of beneficial taxa in the relative abundance in OM. Furthermore, we identified a significant correlation between viral-induced immune responses and microbiome homeostasis in the FC, underscoring its key role in OM mucosal immunity and microbiota homeostasis. Overall, our findings suggest that the defense against pathogenic infections and the maintenance of microbiota homeostasis in vertebrate OM represent an ancient association that predates the emergence of tetrapods.

## Materials and methods

### Fish

All experimental rainbow trout (~ 10 g, 5–6 months old) were sourced from a fish farm in Chengdu (Sichuan, China) and acclimatized in a 16 °C recirculating aquaculture system for 2 weeks. Japanese pufferfish (*Takifugu rubripes*), largemouth bass (*Micropterus salmoides*), common pleco (*Hypostomus plecostomus*), and common carp (*Cyprinus carpio*) were purchased from an aquatic product market in Wuhan (Hubei, China).

### IHNV infection

Healthy rainbow trout were randomly divided into the control group and the infection group. Trout (infection group) were infected with a dose of 2 mL IHNV (1 × 10^7^ TCID_50_) diluted in 10 L aquatic water for 4 h at 16 ℃, and trout (control group) were exposed to virus-free cell culture supernatant and treated the same as the infection group. Then, the trout from both groups were transferred into the new tanks with fresh water and kept for 30 days. Trout were anesthetized with MS-222 before sampling and tissues were collected at 0.5, 1, 4, 7, 14, 21, and 28 DPI.

### Light microscopy and immunofluorescence microscopy studies

The ocular tissues of rainbow trout, Japanese pufferfish, largemouth bass, common pleco (*Hypostomus plecostomus*), and common carp were fixed overnight at 4 °C in 4% neutral buffered formalin. After fixation, the tissues were dehydrated in graded ethanol, embedded in paraffin, and sectioned into 5 μm slices. These slices were stained with H&E and AB according to previously established methods [71]. For detection of IHNV in the trout OM, the sections were incubated overnight at 4 °C with 2 μg/mL mouse anti-IHNV-*N* mAbs (Bio-X Diagnostics, Rochefort, Belgium), followed by 2 μg/mL Cy3 goat anti-mouse IgG for 30 min. Nuclei were stained with DAPI (Invitrogen, Carlsbad, CA, USA) for 8 min before mounting. Cytospin preparations were stained with a Wright-Giemsa stain kit (Thermo Fisher Scientific, Wilmington, DE, USA) according to the manufacturer’s instructions. All sections were observed under an Olympus BX53 microscope (Olympus, Shinjuku City, Tokyo, Japan) and captured with the CellSense Dimension software (Olympus, Shinjuku City, Tokyo, Japan).

### Scanning electron microscopy and transmission electron microscopy (TEM)

For scanning electron microscopy, the fresh and entire ocular tissue of trout was rapidly harvested within 1–3 min using sharp scissors, followed by fixing the tissue with electron microscopy fixative for 2 h. Then the fixed tissues were rinsed with 0.1 M phosphate buffer (PB) (PH 7.4) three times for 15 min each. After that, transfer tissue blocks into 1% OsO4 in 0.1 M PB for 2 h and rinsed again three times by 0.1 M PB (PH7.4) for 15 min each. Next, the tissues were placed in 30%, 50%, 70%, 80%, 90%, 95%, 100%, 100% ethanol for 15 min each, and in isoamyl acetate for 15 min, and finally the tissues were dried in a Critical Point Dryer. Before testing, the tissue samples were sputter-coated with gold for 30 s and then scanned using scanning electron microscopy. For TEM, the tissue samples were fixed by incubated in 30%, 50%, 70%, 80%, 95%, 100%, and 100% ethanol for 20 min each, and finally in acetone twice for 15 min each. They were inserted into the embedding models and polymerized in an oven at 60 °C for 48 h. Subsequently, the resin blocks were ultrathin sliced and transferred onto the 150 meshes cuprum grids, followed by staining with 2% uranium acetate and 2.6% lead citrate for 8 min, respectively. After drying with the filer paper, the cuprum grids were transferred into the grids board to dry overnight. Finally, the cuprum grids were observed under a TEM, and images were captured at the Institute of Hydrobiology of the Chinese Academy of Sciences.

### Laser capture microdissection

Ten-μm-thick, sagittally oriented cryosections of trout ocular tissue from the control, 4 DPI, and 28 DPI groups were prepared as described in a previous study [72]. Briefly, the sections underwent successive immersion in 70% ethanol, ddH_2_O, 75%, 95%, and 100% ethanol for 30 s each, and then immersion in xylene for 5 min. The xylene was allowed to completely evaporate in a sterile fume hood. Next, using a laser capture microdissection (LCM) system, we captured the Cor, BC, and FC regions of the OM. A total of 20 cryosections per trout were used to capture the Cor, BC, and FC samples onto Arcturus Capture Macro LCM caps (Applied Biosystems, Waltham, USA). The captured samples were then immediately processed to extract total RNA using the ArcturusTM PicoPureTM RNA Isolation Kit (Applied Biosystems, Waltham, USA).

### RNA extraction and qPCR analysis

Total RNA was extracted from trout Cor, BC, and FC using TRIzol reagent (Invitrogen, Carlsbad, CA, USA). RNA concentration was assessed using NanoDrop® ND-2000 spectrophotometer (Thermo Fisher Scientific, Wilmington, DE, USA), whereas integrity was determined through agarose gel electrophoresis. Subsequently, about 1 μg of total RNA was used to synthesize cDNA by the Hifair III First-Strand Synthesis System (YEASEN, Shanghai, China). The obtained cDNA was diluted to equal concentrations and used as a template for qPCR analysis. The qPCR program consisted of a first step at 95 °C for 5 min, followed by 40 cycles at 95 °C for 10 s, and 58 °C for 30 s. To determine the viral load, an IHNV plasmid standard curve was constructed, and the IHNV copy number was calculated by extrapolating the mean of each gene copy number from the standard curve. The primers used for qPCR are provided in Table S1.

### Flow cytometry analysis

The proportion of bacteria in the skin, gills, gut, Cor, BC, and FC was determined through flow cytometry analysis. Briefly, the collected tissues were homogenized in sterile phosphate-buffered saline (PBS) and passed through 100 μm cell strainers (SPL Life Sciences, Gyeonggi-do, Republic of Korea). After that, the suspension was centrifuged twice at 400 × *g* for 6 min at 4 °C to discard cells and debris, followed by centrifugation at 16,000 × *g* for 10 min at 4 °C to collect the precipitation (containing bacteria). Finally, the microbiota of Cor, BC, and FC were collected and resuspended with 200 μL PBS, respectively. For bacterial count, the suspension of equal volume (10 μL) was analyzed, and the number of bacteria labeled with SYTO BC by CytoFLEX LX flow cytometer was counted (Beckman Coulter, Brea, CA, USA). To determine the proportion of lymphocytes and myeloid cells in the skin, gills, gut, Cor, BC, and FC, the tissues were homogenized in DMEM (Gibco™, Gaithersburg, MD, USA) supplemented with 1% FBS and filtered by 100 μm cell strainers. The obtained cell suspensions were layered onto a 34 to 51% Percoll (Cytiva, Uppsala, Sweden) discontinuous density gradient and centrifuged at 400 × *g* for 30 min at 4 °C. The leukocytes at the interface were collected and then washed with DMEM twice for 6 min each. the proportion of lymphocytes and myeloid cells was determined based on FSC and SSC signals detected by CytoFLEX LX flow cytometer (Beckman Coulter, Brea, CA, USA).

### Antibiotics treatment

The method for antibiotics was used as previous studies reported with slightly modified [73]. Rainbow trout were exposed to a mixture of the 6 antibiotics (including Amoxicillin crystalline, Kanamycin sulfate, Erythromycin, Enrofloxacin, oxytetracycline dihydrate, and Doxycycline hydrochloride, 25 mg/L for each antibiotic) for 4 days. Exposure solutions were renewed daily to maintain the appropriate concentration of antibiotics. To detect the microbial changes in trout OM after treatment, Cor, BC, and FC tissue were collected, weighed, and homogenized in 1 ml PBS (filtered with 0.22 μm) for coating plates to calculate the number of colonies.

### RNA-Seq library and data analyses

The Cor, BC, and FC samples from the control group and the IHNV-infected group at 4 and 28 DPI were sent to Majorbio Bio-Pharm Technology Co. Ltd. (Shanghai, China) for RNA-Seq analysis. RNA-Seq libraries were prepared and analyzed as we previously described [29]. The prepared libraries were then sequenced using the Illumina HiSeq X Ten/NovaSeq 6000 sequencer to generate paired-end reads with a length of 150 bp. The raw reads were trimmed and quality-controlled using SeqPrep and Sickle, and the remaining clean reads were mapped to the genome assembly of *Oncorhynchus mykiss*. Genes were considered as the differentially expressed genes (DEGs) if false discovery rate (FDR) ≤ 0.05 and |log2 (fold-change) |≥ 1.

### Analysis of bacterial translocation in trout OM

For microbiota detection of the trout Cor, BC, and FC, fluorescent in situ hybridization was applied as previously described [74]. Cryosections (10 µm) of ocular tissue smears from control trout were fixed in 4% PFA for 10 min and stained with Cy3-labeled EUB338 (anti-sense probe) and Cy3-labeled NONEUB oligonucleotide probes at their 5’ ends. Hybridizations were conducted in 2 × SSC/10% formamide (hybridization buffer) with 1 μg/mL of the labeled probes for 14 h at 37 °C. Subsequently, the slides were washed with hybridization buffer without probes, washing buffer (2 × SSC), and PBS each two times at 37 °C. Nuclei were stained with DAPI (Invitrogen, Carlsbad, CA, USA) for 8 min before mounting. All sections were observed under an Olympus BX53 microscope and captured with the CellSense Dimension software (Olympus, Shinjuku City, Tokyo, Japan).

### qPCR to determine bacterial abundance in trout tissues

Total genomic DNA was isolated from Cor, BC, FC, skin, gills, and gut tissues using the QIAamp DNA Mini Kit (Qiagen, Hilden, Germany). Absolute qPCR analysis was conducted to estimate the copy numbers of total bacteria using V3–V4 region-specific primers shown in Table S1. In brief, pMD 19-T vector containing the 16S rRNA gene V3–V4 fragments insert was prepared from recombinant DH5α Escherichia coli cells. Plasmid DNA was isolated from an overnight selective culture using HiPure Plasmid Micro Kit (OMEGA, Norcross, USA). Standard curves were generated by serial dilutions of known copy numbers of plasmids containing 16S rRNA gene V3-V4 fragments [75]. The Ct values of the samples were extrapolated into the standard curve to calculate the copy number. qPCR was conducted in a 20-μL reaction mixture containing 10 μL 2 × SYBR Green qPCR Mix (YEASEN, Shanghai, China), 0.5 μL of each primer (10 μM), 1.0 μL of 100 ng DNA templates, and 8.0 μL of nuclease-free water. The qPCR program consisted of an initial step at 95 °C for 5 min, followed by 40 cycles at 95 °C for 10 s, and 58 °C for 30 s. All samples were analyzed in triplicate to ensure accuracy.

### Bacterial 16S rRNA sequencing and data analyses

Purified amplicons were equimolarly pooled, and sequencing was performed using the MiSeq platform with the MiSeq Reagent Kit v3 to generate paired-end reads with a length of 250 bp (Illumina, China). Sequence data analysis was conducted using QIIME2 and R packages [76]. Raw reads were de-multiplexed using the demux plugin and then filtered, denoised, merged, and chimera-removed by DADA2 [77]. Based on SILVA database (Release 132), taxonomy was assigned to amplicon sequence variants (ASVs) using the classify-sklearn naïve Bayes taxonomy classifier in the feature-classifier plugin [78]. Finally, the diversity plugin was used to estimate alpha- (Chao1 and Shannon) and beta (weighted UniFrac) diversity metrics. LEfSe analysis was performed to detect differentially abundant taxa across groups using default parameters [79].

### Statistics

Unpaired Student’s *t* test (Prism version 8.0; GraphPad) and one-way analysis of variance with Bonferroni correction were used to evaluate the differences between the OM sites or groups Data are expressed as mean ± SEM. All *p* values < 0.05 were considered statistically significant.

### Supplementary Information

**Additional file 1: Figure S1.** The evolution of the vertebrate ocular mucosa. a–d show the jawless fishes, cartilaginous fishes, teleost fishes, and tetrapods, respectively. **Figure S2.** a–e H&E (upper) and AB (lower) stain of the three distinct regions of OMs from *Oncorhynchus mykiss* (a), *Takifugu rubripes *(b), *Hypostomus plecostomus* (c), *Micropterus Salmoides* (d), and *Cyprinus carpio* (e). From left to right: Cor, BC, and FC. EP, epithelium; ST, stroma; LP, lamina propria. Red arrowheads indicate mucus cells. Black arrowheads indicate indicate lymphocytes. Scale bar, 50 μm. **Figure S3.** Fish species selected from five different families for understanding of the teleost OM. **Figure S4.** a Real-time PCR analysis of bacteria V3-V4 16S rRNA region in the Cor, BC, FC, and other MALTs (i.e., skin, gill, and gut). DNA abundance was normalized to that of the Cor, which is set as 1 (*n* = 9). Statistical differences were evaluated by one-way ANOVA. Data are presented as mean ± SEM of three independent duplicates. *p < 0.05, **p < 0.01, ***p < 0.001. b Dot plots representing the Cor, BC, and FC samples without staining of SYTO BC Green. **Figure S5.** Dot plots representing lymphocytes and myeloid cells in skin, gill, and gut of control trout. **Figure S6.** a Immunofluorescence staining of IHNV in the Cor, BC, and FC paraffin-sections from 4, and 28 days infected and control trout. From left to right: Cor, BC, and FC. EP, epithelium; ST, stroma; LP, lamina propria. IHNV were stained red with an anti-IHNV-*N* mAb, while nuclei were stained blue with DAPI. Scale bars, 20 μm. b Numbers of virus-infected cells were counted from a (*n* = 9). c H&E stain of the three distinct regions of control and IHNV-infected fish OM at 4 and 28 DPI. From left to right: Cor, BC, and FC. EP, epithelium; ST, stroma; LP, lamina propria. Red arrowheads show disrupted mucosal EP with loss of continuity. Scale bar, 20 μm. d Pathology score of the Cor, BC, and FC of control and IHNV-infected trout at 4 and 28 DPI (*n* = 9). One-way ANOVA test was used to evaluate the statistical differences. Data are presented as mean ± SEM of three biological duplicates. ***p < 0.001. **Figure S7.** Immunofluorescence staining of IHNV with isotype control antibodies for anti-IHNV-N with in the Cor, BC, and FC paraffin-sections. Scale bars, 20 μm. **Figure S8.** Altered KEGG pathways in the Cor, BC, and FC at 4 and 28 DPI versus control trout detected by RNA-Seq. **Figure S9.** a–c Bar chart showing the log-transformed LDA values of bacterial taxa significantly correlated with trout infected with IHNV and control group at 28 DPI in the Cor (a), BC (b), and FC (c) by LEfSe (*p* < 0.05). **Table S1.** Primers used in this study.

## Data Availability

The raw 16S RNA sequencing data and RNA-seq data have been deposited in the NCBI Sequence Read Archive under BioProject accession numbers PRJNA1006525 and PRJNA1008220, respectively.
