# Correction: Ocular mucosal homeostasis of teleost fish provides insight into the coevolution between microbiome and mucosal immunity

**DOI:** 10.1186/s40168-024-01767-3

**Published:** 2024-02-08

**Authors:** Weiguang Kong, Gaofeng Cheng, Jiafeng Cao, Jiaqian Yu, Xinyou Wang, Zhen Xu

**Affiliations:** 1 Key Laboratory of Breeding Biotechnology and Sustainable Aquaculture, Institute of Hydrobiology, Chinese Academy of Sciences, Wuhan, 430072 China; 2 Department of Aquatic Animal Medicine, College of Fisheries, Huazhong Agricultural University, Wuhan, 430070 Hubei China; 3 Laboratory of Biochemistry and Molecular Biology, School of Marine Sciences, Meishan Campus, Ningbo University, Ningbo, 315832 China; 4 Liaoning Key Laboratory of Marine Animal Immunology, Dalian Ocean University, Dalian, 116023 China

**Correction: Microbiome 12, 10 (2024)**

**https://doi.org/10.1186/s40168-023-01716-6**

Following publication of the original article [1], the author reported that Figures 3,4,5 and 9 have a yellow background and should be removed. Moreover, in the videobyte, the word „oral “ should be „ocular “ in the very first sentence of the videobyte.

The original article has been updated.
