# Supplementary material for: Ocular mucosal homeostasis of teleost fish provides insight into the coevolution between microbiome and mucosal immunity
Source: Microbiome. 2024 Jan 13;12:10. doi: 10.1186/s40168-023-01716-6 (PMC10787490; doi:10.1186/s40168-023-01716-6)
Supplement: Supplementary file 2 — Additional file 1: Figure S1. The evolution of the vertebrate ocular mucosa. a–d show the jawless fishes, cartilaginous fishes, teleost fishes, and tetrapods, respectively. Figure S2. a–e H&E (upper) and AB (lower) stain of the three distinct regions of OMs from Oncorhynchus mykiss (a), Takifugu rubripes (b), Hypostomus plecostomus (c), Micropterus Salmoides (d), and Cyprinus carpio (e). From left to right: Cor, BC, and FC. EP, epithelium; ST, stroma; LP, lamina propria. Red arrowheads indicate mucus cells. Black arrowheads indicate indicate lymphocytes. Scale bar, 50 μm. Figure S3. Fish species selected from five different families for understanding of the teleost OM. Figure S4. a Real-time PCR analysis of bacteria V3-V4 16S rRNA region in the Cor, BC, FC, and other MALTs (i.e., skin, gill, and gut). DNA abundance was normalized to that of the Cor, which is set as 1 (n = 9). Statistical differences were evaluated by one-way ANOVA. Data are presented as mean ± SEM of three independent duplicates. *p < 0.05, **p < 0.01, ***p < 0.001. b Dot plots representing the Cor, BC, and FC samples without staining of SYTO BC Green. Figure S5. Dot plots representing lymphocytes and myeloid cells in skin, gill, and gut of control trout. Figure S6. a Immunofluorescence staining of IHNV in the Cor, BC, and FC paraffin-sections from 4, and 28 days infected and control trout. From left to right: Cor, BC, and FC. EP, epithelium; ST, stroma; LP, lamina propria. IHNV were stained red with an anti-IHNV-N mAb, while nuclei were stained blue with DAPI. Scale bars, 20 μm. b Numbers of virus-infected cells were counted from a (n = 9). c H&E stain of the three distinct regions of control and IHNV-infected fish OM at 4 and 28 DPI. From left to right: Cor, BC, and FC. EP, epithelium; ST, stroma; LP, lamina propria. Red arrowheads show disrupted mucosal EP with loss of continuity. Scale bar, 20 μm. d Pathology score of the Cor, BC, and FC of control and IHNV-infected trout at 4 and 28 DPI (n = 9) [file 40168_2023_1716_MOESM1_ESM.docx]

**Supplementary Information**

Ocular mucosal homeostasis of teleost fish provides insight into the coevolution between microbiome and mucosal immunity

Weiguang Kong, Gaofeng Cheng, Jiafeng Cao, Jiaqian Yu, Xinyou Wang & Zhen Xu


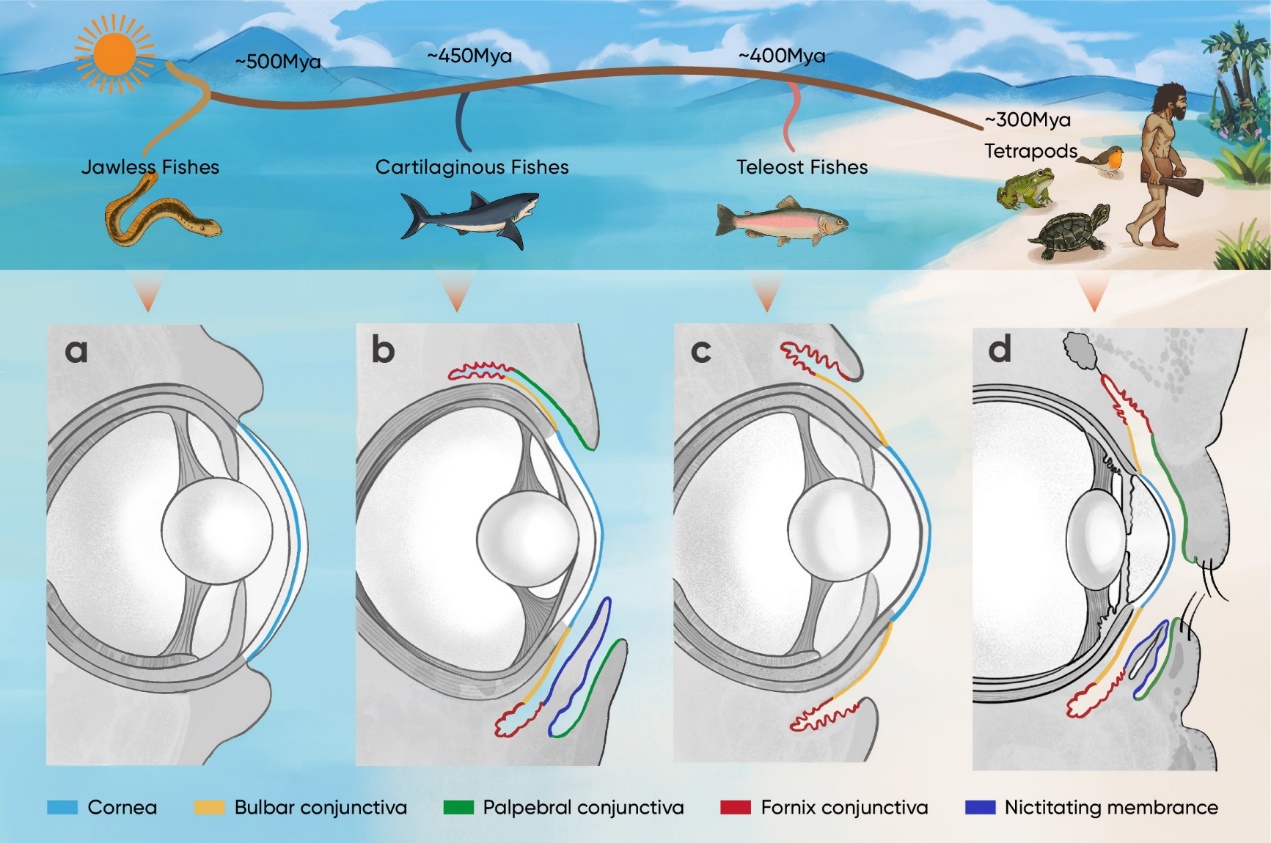


Figure S1. The evolution of the vertebrate ocular mucosa. **a**–**d** show the jawless fishes, cartilaginous fishes, teleost fishes, and tetrapods, respectively.


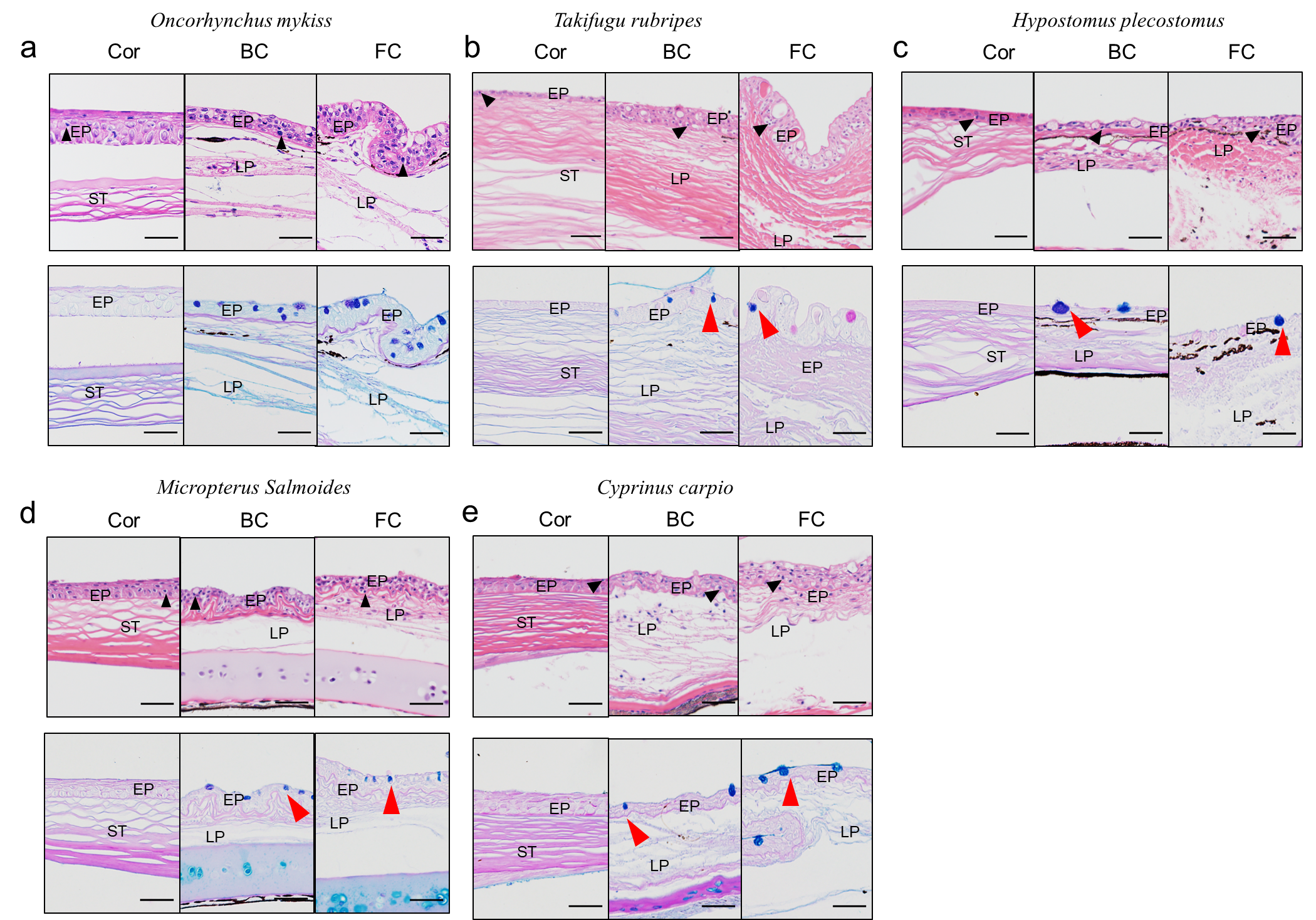


**Figure S2. a**–**e** H&E (upper) and AB (lower) stain of the three distinct regions of OMs from *Oncorhynchus mykiss* (**a**), *Takifugu rubripes* (**b**), *Hypostomus plecostomus* (**c**), *Micropterus Salmoides* (**d**), and *Cyprinus carpio* (**e**). From left to right: Cor, BC, and FC. EP, epithelium; ST, stroma; LP, lamina propria. Red arrowheads indicate mucus cells. Black arrowheads indicate lymphocytes. Scale bars, 50 μm.


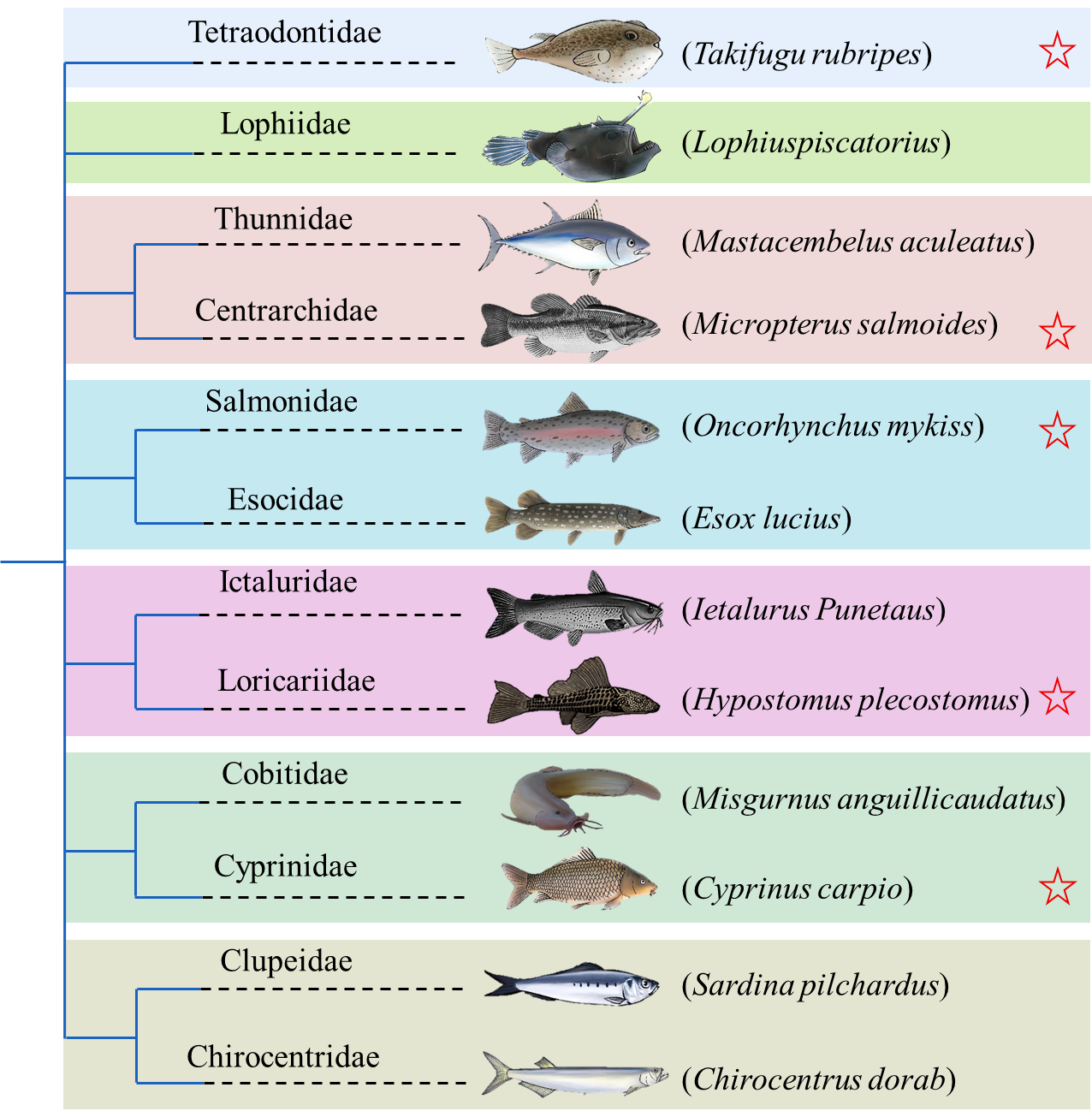


**Figure S3.** Fish species selected from five different families for understanding of the teleost OM.


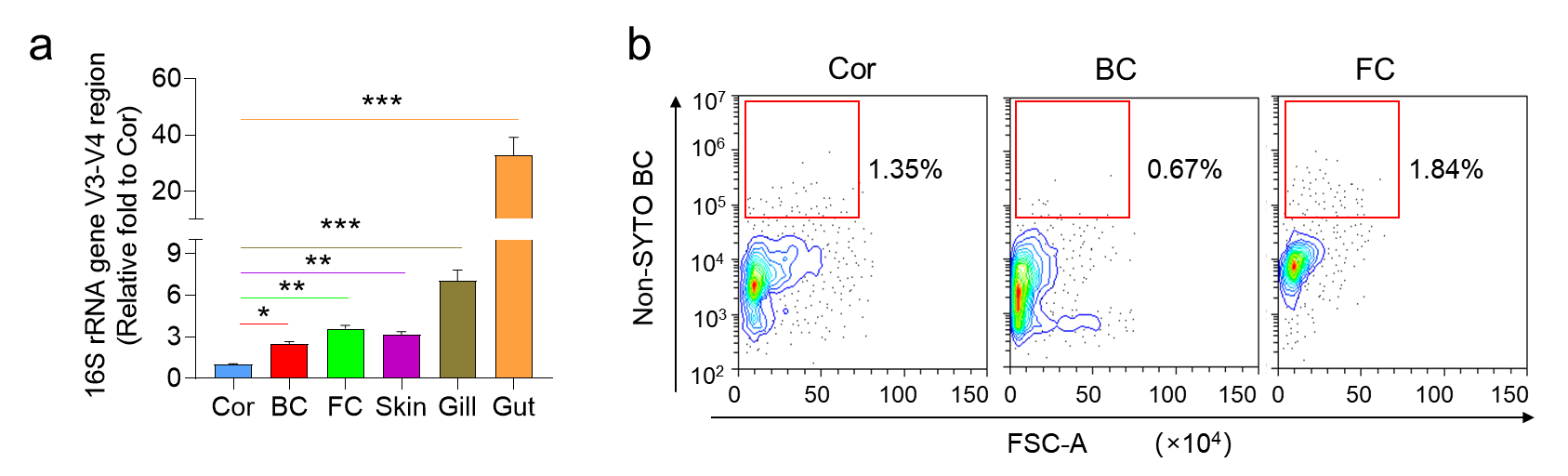


**Figure S4. a** Real-time PCR analysis of bacteria V3-V4 16S rRNA region in the Cor, BC, FC, and other MALTs (i.e., skin, gill, and gut). DNA abundance was normalized to that of the Cor, which is set as 1 (*n* = 9). Statistical differences were evaluated by one-way ANOVA. Data are presented as mean ± SEM of three independent duplicates. **p* < 0.05, ***p* < 0.01, ****p* < 0.001. **b** Dot plots representing the Cor, BC, and FC samples without staining of SYTO BC Green.

**
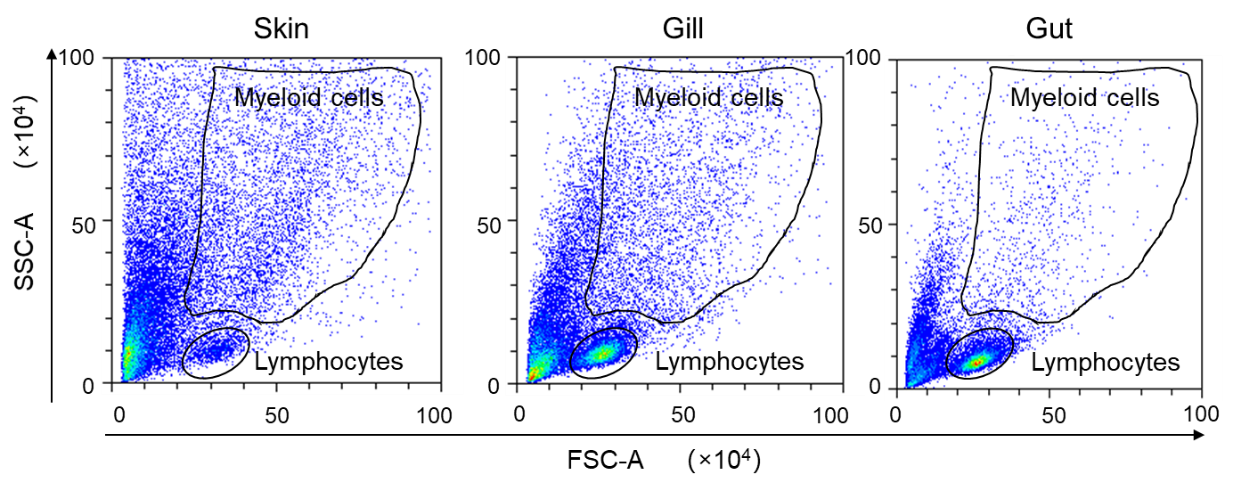
**

**Figure S5.** Dot plots representing lymphocytes and myeloid cells in skin (**a**), gill (**b**), and gut (**c**) of control trout.

**
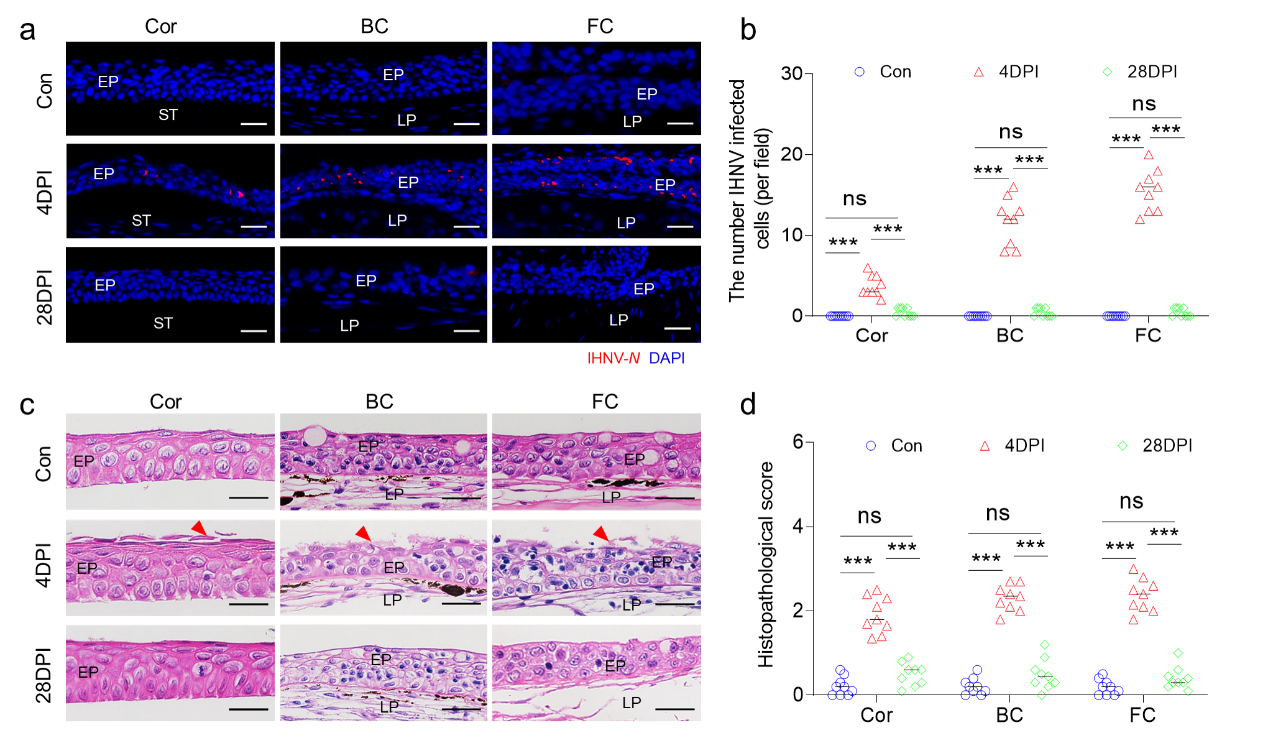
**

**Figure S6. a** Immunofluorescence staining of IHNV in the Cor, BC, and FC paraffin-sections from 4, and 28 days infected and control trout. From left to right: Cor, BC, and FC. EP, epithelium; ST, stroma; LP, lamina propria. IHNV were stained red with an anti-IHNV-*N* mAb, while nuclei were stained blue with DAPI. Scale bars, 20 μm. **b** Numbers of virus-infected cells were counted from **a** (*n* = 9). **c** H&E stain of the three distinct regions of control and IHNV-infected fish OM at 4 and 28 DPI. From left to right: Cor, BC, and FC. EP, epithelium; ST, stroma; LP, lamina propria. Red arrowheads show disrupted mucosal EP with loss of continuity. Scale bar, 20 μm. **d** Pathology score of the Cor, BC, and FC of control and IHNV-infected trout at 4 and 28 DPI (*n* = 9). One-way ANOVA test was used to evaluate the statistical differences. Data are presented as mean ± SEM of three biological duplicates. ****p* < 0.001.


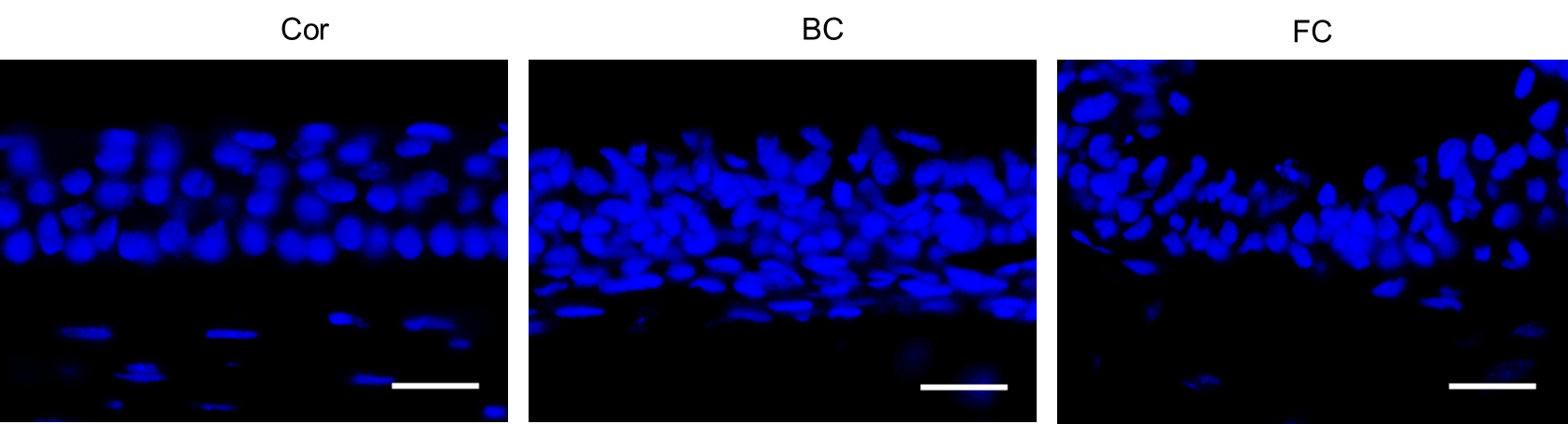


**Figure S7.** Immunofluorescence staining of IHNV with isotype control antibodies for anti-IHNV-*N* with in the Cor, BC, and FC paraffin-sections. Scale bars, 20 μm.
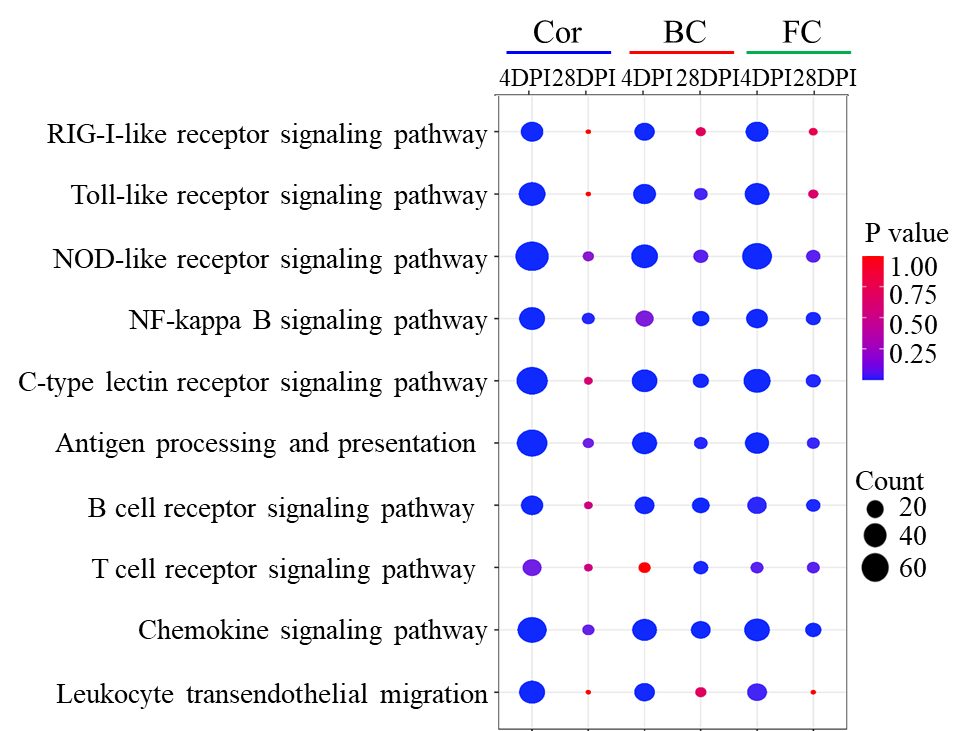


**Figure S8.** Altered KEGG pathways in the Cor, BC, and FC at 4 and 28 DPI versus control trout detected by RNA-Seq.

**
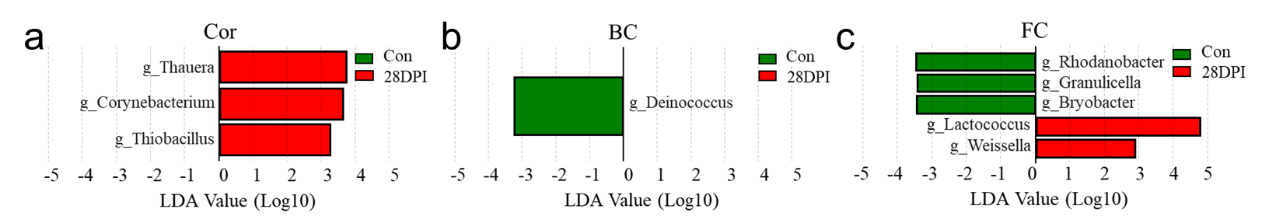
**

**Figure S9. a**–**c** Bar chart showing the log-transformed LDA values of bacterial taxa significantly correlated with trout infected with IHNV and control group at 28 DPI in the Cor (**a**), BC (**b**), and FC (**c**) by LEfSe (*p* < 0.05)

.

**Table S1. Primers used in this study.**

| **Gene** | **Primer Sequence (5’-> 3’)** | |
| --- | --- | --- |
|  | **Forward primer** | **Reverse primer** |
| *cd80/86* | CATCCAGAGACTCCCAAACCA | GGATGGTAGGTCCCTCGTCTT |
| *lyz* | AGCCGCTACTGGTGTGACGA | CTGACAGTGAAGGCGCCAAG |
| *cd22* | TGAAGATGACAGTGGCAGAT | GGAGGGTTACAGGTGGAG |
| *igt* | CAGACAACAGCACCTCACCTA | GAGTCAATAAGAAGACACAACGA |
| *igm* | AAGAAAGCCTACAAGAGGGAGA | CGTCAACAAGCCAAGCCACTA |
| *igd* | CAGGAGGAAAGTTCGGCATCA | CCTCAAGGAGCTCTGGTTTGGA |
| *ccr10* | ATCTGATGACTGTTGTTG | GTGGAAGTGTCGTTGTAG |
| *ccl20b* | GGCTGCTCTGTTCTCTCT | CTCTTGGTCCAGTCTTGA |
| *ccl9* | GGATTGCTGTCTCCTCAC | CACCCACTTGCTCTTTGT |
| *cxcl13* | CAGGTTTCACAAGTTGGAGA | TCCCTTGTTGTTACTTTGTAAC |
| *cd4-1* | TGGTCGAGAGACGATAGATCC | GAGGTACTTGTTTGTGGCATGA |
| *cd4-2a* | CGTGAGAAGTTTGTTGCCGAA | TGGCTGCCTTTGGTACAGTGA |
| *cd4-2b* | AAGCCCCTCTTGCCGAGGAA | CTCAACGCCTTTGGTACAGTGA |
| *tcr-α* | CAGCTTGAAGTCAAGAAATAC | TATCAGCACGTTGAAAACGAT |
| *tcr-β* | CTCCGCTAAGGAGTGTGAAGATAG | CAGGCCATAGAAGGTACTCTTAGC |
| *cd8α* | ACTGCCAAGTCGTGCAAAGTG | AAGCCACAGCCAGCAGTCAA |
| *cd8β* | TCCTGTATGCTCCAGAACCAG | ATGTTGGGCGAGTTTCTCCG |
| *cd209* | GCCGTGTGACCACATCTACA | CGAGATGAGCGGGAAGTTCA |
| *cd11b* | AACCACAGCAGTGTTCACCA | TTCCGTTGTCACTCAGTCCG |
| *ccl25* | GTTCTTTCTCCTGCTCCT | ACAGTCTTCCGTTTCTTC |
| *mrc1* | GGCATAAGAACCCGGTCACA | ATTGAGGCCAAGTCTGCTCC |
| *ncf2* | TTCTCTGGCTTTGCTCCGTT | AGACGATATTGCCAGGCACC |
| *mpeg1* | CTCAGACGTGTCCTTCCTCTC | CGTGTATAAGAAGTTACGCACTTG |
| *mpo* | ACGGACGTGAGTTTCTTCCC | ACGGACGTGAGTTTCTTCCC |
| *rig-i* | CAGAGGTACTACAGGAAATGG | TTACTGGTCTTCAAGCAATG |
| *mda5* | CAGTGGAGATGACGATGGG | ACTTGGCGTTCTTGTGCTT |
| *mx1* | GATGCTGCACCTCAAGTCCTACTA | CGGATCACCATGGGAATCTGA |
| *lgp2* | AGTTTGGCACGCAGGAGTA | CAAGCAGGAAGAAGTCGGT |
| *ifn-γ* | CTGAAAGTCCACTATAAGATCTCCA | CCCTGGACTGTGGTGTCAC |
| *Il-6* | GCTCGGGACAGACGGTGAAA | GAAACTCCTCCACAAACTGTTG |
| *Il-8* | GGCCCTCCTGACCATTACTGA | TCCAGACAAATCTCCTGACCG |
| *Il-10b* | GAGCTGGAGACACCTCTTCTC | TAAAGTTGTGTGTTCCATTGTTG |
| *Il-1β* | ACCGAGTTCAAGGACAAGGA | CATTCATCAGGACCCAGCAC |
| *saa* | TTGTTCTGACCCTCGTTG | CCTGGCAGCATCATAGTT |
| *ef-1a* | CAACGATATCCGTCGTGGCA | ACAGCGAAACGACCAAGAGG |
| *hp1* | CGGAGGAGGTTGGAAGC | GCAGCAGAAGCCACAGC |
| *cath1* | CTGGAGGCAAGCAACAAC | CCCCCAAGACGAGAGACA |
| *c3* | GAGATGGCCTCCAAGAAGATAGAA | ACCGCATGTACGCATCATCA |
| *cd209* | GCCCTCTTTGTGCTGATT | ACTCCCTACACTTCCTTA |
| *lyz2* | TCTCCTGCTTGTGGCTGTG | TCGGTGTTGCGGTTGGTG |
| *lect2* | GAAGGCAGCCATAAACGA | ATCCCTGGGTAGGCACTT |
| *nox1* | CACCAATCCACCACATAA | AGGAAGACATACTGACCC |
| *cmpk2* | AAAGAGTTACCTCCAGAATG | CGCACAAGTAGGAGCATA |
| IHNV-*N* | TTAACTTCAACGCCAACAGG | TCGGACAGGTTGATGAGAATG |
| 16S V3-V4 | ACTCCTACGGGAGGCAGCA | GGACTACHVGGGTWTCTAAT |
